# Transcriptome-wide mapping of N^3^-methylcytidine modification at single-base resolution

**DOI:** 10.1093/nar/gkaf153

**Published:** 2025-03-12

**Authors:** Yunyi Gao, Jingyu Hou, Saisai Wei, Canlan Wu, Sujun Yan, Jia Sheng, Jun Zhang, Zhanghui Chen, Xiangwei Gao

**Affiliations:** Department of Clinical Laboratory of Sir Run-Run Shaw Hospital, and School of Public Health, Zhejiang University School of Medicine, Hangzhou 310058, China; Department of Clinical Laboratory of Sir Run-Run Shaw Hospital, and School of Public Health, Zhejiang University School of Medicine, Hangzhou 310058, China; Zhanjiang Institute of Clinical Medicine, Zhanjiang Central Hospital, Zhanjiang 524000, China; Key Laboratory of Laparoscopic Technology of Zhejiang Province, Department of General Surgery, Sir Run-Run Shaw Hospital, Zhejiang University School of Medicine, Hangzhou 310016, China; Department of Clinical Laboratory of Sir Run-Run Shaw Hospital, and School of Public Health, Zhejiang University School of Medicine, Hangzhou 310058, China; Department of Clinical Laboratory of Sir Run-Run Shaw Hospital, and School of Public Health, Zhejiang University School of Medicine, Hangzhou 310058, China; Department of Chemistry, The RNA Institute, University at Albany SUNY, Albany, NY 12222, United States; Department of Clinical Laboratory of Sir Run-Run Shaw Hospital, and School of Public Health, Zhejiang University School of Medicine, Hangzhou 310058, China; Zhanjiang Institute of Clinical Medicine, Zhanjiang Central Hospital, Zhanjiang 524000, China; Department of Clinical Laboratory of Sir Run-Run Shaw Hospital, and School of Public Health, Zhejiang University School of Medicine, Hangzhou 310058, China

## Abstract

3-Methylcytidine (m^3^C), a prevalent modification of transfer RNAs (tRNAs), was recently identified in eukaryotic messenger RNAs (mRNAs). However, its precise distribution and formation mechanisms in mRNAs remain elusive. Here, we develop a novel approach, m^3^C immunoprecipitation and sequencing (m^3^C-IP-seq), utilizing antibody enrichment to profile the m^3^C methylome at single-nucleotide resolution. m^3^C-IP-seq captures 12 cytoplasmic tRNA isoacceptors and 2 mitochondrial tRNA isoacceptors containing m^3^C modifications. Moreover, m^3^C-IP-seq permits the comprehensive profiling of m^3^C sites in mRNAs and long noncoding RNAs, with their presence reliant on a nuclear isoform of METTL8. A significant proportion of m^3^C sites is concentrated in the 3′ untranslated region (3′ UTR) of mRNAs and is associated with mRNA degradation. Additionally, m^3^C methylation is dynamic and responds to hypoxia. Collectively, our data demonstrate the widespread presence of m^3^C modification in the human transcriptome and provide a resource for functional studies of m^3^C-mediated RNA metabolism.

## Graphical Abstract

**Graphical Abstract:**
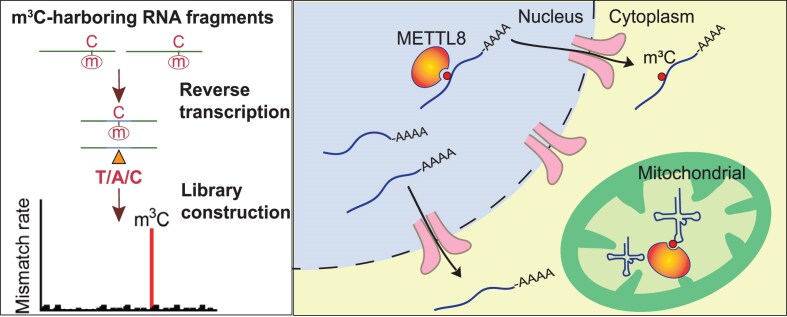

## Introduction

The code generated by RNA modifications embeds an important layer of gene expression regulation [1]. The RNA modifications, which are dynamically controlled by the “writers”, “erasers”, and “readers”, post-transcriptionally influence gene expression by regulating RNA processing, stability, or messenger RNA (mRNA) translation [2]. Recently, the development of detection methods, based on analytical chemistry and high-throughput sequencing, has greatly advanced our understanding of several mRNA modifications such as 6-methyladenosine (m^6^A) [3, 4], 1-methyladenosine (m^1^A) [5–7], 5-methylcytidine (m^5^C) [8], 5-hydroxymethylcytosine (5hmC) [9], 4-acetylcytidine (ac^4^C) [10], and N7-methylguanosine (m^7^G) [11]. However, most of the RNA modifications' distribution and biological function, especially within mRNAs, remain to be explored.

3-Methylcytidine (m^3^C) was first discovered in total RNAs from several species and later found in eukaryotic transfer RNAs (tRNAs), ribosomal RNAs, and mRNAs [12, 13]. A majority of m^3^C modifications are distributed in tRNA_Arg_^CCU^, tRNA_Arg_^UCU^, all tRNA_Ser_, and all tRNA_Thr_ at specific positions [14]. Unbiased mass spectrometry experiments revealed that m^3^C was consistently detected in poly(A) RNA isolated from human cells and mouse tissue at a level comparable to m^5^C and m^1^A [15]. Studies have identified METTL2 (METTL2A and METTL2B in human), METTL6, and METTL8 as the m^3^C methyltransferases. METTL2 and METTL6 catalyze m^3^C formation in tRNAs, while METTL8 contributes to m^3^C formation in mRNAs [15]. However, METTL8 has recently been reported as a mitochondrial protein that installs m^3^C modification on mt-tRNA_Thr_ and mt-tRNA_Ser_^UCN^, regulating mitochondria protein translation and mitochondrial respiratory activity [16–20]. METTL8 influences mitochondrial protein translation and function in cortical neural stem cells by modifying mt-tRNA_Thr/Ser_^UCN^ with m^3^C. Deletion of METTL8 in mice results in rapid depletion of embryonic cortical neural stem cells and increasing neuronal differentiation [21]. Recent studies have reported that ALKBH3 demethylates m^3^C in tRNAs while ALKBH1 demethylates m^3^C in mRNAs [22, 23], implying that m^3^C is dynamically regulated.

m^3^C modification in RNAs induces truncation or misincorporations during complementary DNA (cDNA) synthesis. Based on this feature, several techniques, such as ARM-seq [24], HAMR [25], and DM-tRNA-seq [26], have been developed for the mapping of m^3^C modification. In addition, chemistry-based detecting approaches, such as AlkAniline-seq [27] and hydrazine-aniline cleavage sequencing (HAC-seq) [28], have been developed. The AlkAniline-seq allows simultaneously profile m^3^C and m^7^G modifications [27], while HAC-seq specifically maps m^3^C sites in tRNAs at single-base resolution [28]. However, due to the relatively low abundance of m^3^C, mapping m^3^C modification in mRNAs remains challenging.

Here, we present m^3^C-methylated RNA immunoprecipitation sequencing (m^3^C-IP-seq) to transcriptome-wide map m^3^C sites in tRNAs, mRNAs, and long noncoding RNAs (lncRNAs). We also identify the nucleus-localizing isoform of METTL8 as a methyltransferase that contributes to a subset of m^3^C within mRNAs and lncRNAs. Our study reveals that m^3^C modification is mainly distributed in the 3′ UTR regions of mRNAs and is associated with mRNA degradation. Moreover, we show that this modification is dynamically regulated by hypoxia, highlighting its crucial role in post-transcriptional regulation and cellular stress responses.

## Materials and methods

### Cell culture

HEK293T cells and HCT116 cells were obtained from ATCC and cultured in DMEM medium (Gibco) supplemented with 10% fetal bovine serum (Thermo Fisher Scientific). Cells were maintained at 37°C in a 5% CO_2_ atmosphere.

### RNA isolation

Total RNA was isolated from cells using TRIzol reagent (Life Technologies) following the manufacturer’s instructions. tRNA was purified by 7 M 10% denaturing polyacrylamide Tris-Borate-EDTA–urea gel (TBE–urea gel). poly(A) RNA was purified using Dynabeads Oligo(dT)_25_ (Invitrogen).

### Quantitative reverse transcriptase-polymerase chain reaction (RT-qPCR)

Total RNA was isolated from cells using TRIzol reagent (Life Technologies). Each RNA sample was reverse transcribed with the M-MLV reagent (Takara) using random primers. Real-time polymerase chain reaction (PCR) was performed using the SYBR Green I master mix on a LightCycler 480 real-time PCR system (Roche). Relative RNA expression was normalized to either *ACTB* or *GAPDH* and calculated using the 2^(−ΔΔCT)^ method. RT-qPCR primers are listed in Supplementary Table S1.

### Plasmids construction

Genes encoding different METTL8 (NCBI: 79 828) isoforms were amplified from HEK293T cell line cDNA and cloned into vector pCDH-CMV-MCS-EF1-Puro with a C-terminal EGFP tag. The initiating Met codon (ATG) of EGFP was mutated to the Ile codon (ATA) to prevent the potential generation of free EGFP. METTL8 isoforms were also cloned into vector pCDH-CMV-MCS-EF1-Puro with a C-terminal FLAG tag. For CRISPR/Cas9 constructs, guide sequence targeting exon 6 of the conserved *METTL8* open reading frame was cloned into the vector pX458M (Addgene). The primers used are listed in Supplementary Table S1.

### Immunoblotting

Cells were lysed in RIPA lysis buffer (Beyotime). Forty micrograms of total protein was separated by sodium dodecyl sulfate-polyacrylamide gel (SDS-PAGE) under denaturing conditions and transferred to polyvinylidene fluoride (PVDF) membranes (Millipore). Membranes were blocked in 5% nonfat dry milk in Tris-Buffered Saline with Tween 20 (TBST) and incubated with primary antibodies, followed by incubation with the secondary antibody and chemiluminescent detection system (Bio-Rad). The primary antibodies are listed in Supplementary Table S2.

### Fluorescence assay

Cells expressing EGFP-tagged METTL8 were treated with Mito-Tracker Red CMXRos (Beyotime) at 37°C for 30 min, washed three times in phosphate-buffered saline (PBS), and fixed in 4% paraformaldehyde for 30 min. The fixed cells were immunolabeled with the nuclear counterstain Hoechst (Thermo Fisher Scientific) in PBS for 5 min at room temperature. Fluorescent images were captured using a Nikon A1R confocal microscope and analyzed.

### m^3^C dot blot

Equal RNA samples were spotted to a Hybond-N + membrane (GE Healthcare) and crosslinked with ultraviolet light at 254 nm (0.12 J/cm^2^). After blocking in PBS containing 5% nonfat milk and 0.05% Tween-20 for 1 h, the membrane was incubated with the anti-m^3^C antibody at room temperature for 4 h. The membrane was incubated with horseradish peroxidase-conjugated anti-rabbit IgG at room temperature for 1 h and visualized using enhanced chemiluminescence. For the competition assay, the anti-m^3^C antibody was pre-mixed with increasing concentrations of 3-methylcytidine or cytosine for 1.5 h at room temperature before applying it to the crosslinked tRNA membrane.

### m^3^C-IP-seq

Dynabeads™ Protein G beads (Invitrogen) were coated with 6 μg of anti-m^3^C antibody in an immunoprecipitation buffer for 30 min. For immunoprecipitation, 20 μg of tRNA or fragmented poly(A) RNA was incubated with antibody-coated Protein G beads at 4°C for 4 h. The immunoprecipitation complex was digested with Proteinase K (NEB) at 55°C for 1 h, followed by RNA extraction. Half of the immunoprecipitated m^3^C-containing RNA fragments were treated with AlkB demethylase followed by RNA extraction.

tRNA or fragmented poly(A) RNA (as “Input”), immunoprecipitated RNA (as “IP”), and demethylated immunoprecipitated RNA (as “Dm”) were subjected to library construction. To remove 3′ phosphorylation groups, RNA samples were incubated at 37°C for 1 h with PNK (NEB). The dephosphorylated RNA was ligated to 1 μl of 20 μM 3′ RNA adaptor catalyzed by T4 RNA ligase 2, truncated (NEB) at 25°C for 2 h. Following ligation, cDNA synthesis began with hybridization of the 1 μl of 20 μM RT primer to the ligated RNA. Synthesis of cDNA was carried out using Superscript IV (Invitrogen) at 55°C for 1 h. Template RNA was degraded by adding 1 μl of 5 M NaOH at 95°C for 3 min, followed by neutralization with 1 μl of 5 M HCl. RT product was purified by 7 M 12% denaturing polyacrylamide TBE–urea gel. Ligation of the cDNA and 5′ DNA adaptor was performed with T4 RNA ligase 1, high concentration (NEB) at 0.5 U/μl at 25°C overnight. The ligated cDNA was purified using 7 M 12% denaturing polyacrylamide TBE–urea gel and then was amplified using the NEB Next^®^ High-Fidelity 2 × PCR Master Mix (NEB) with universal and index primers. The libraries were sequenced on an Illumina NovaSeq 6000 with paired-end 2 × 150 bp read length. The adaptors and primers are listed in Supplementary Table S1.

### Quantitative validation of m^3^C-IP-seq

A 31-bp oligonucleotide with the 11th position cytidine modified with m^3^C was synthesized as described previously [29]. An identical sequence without m^3^C modification was used as non_m^3^C control. The sequences are detailed in Supplementary Table S1. These oligonucleotides were mixed at varying molar ratios and subjected to m^3^C-IP-seq. Additionally, 20 μg of tRNA was mixed with a 10% m^3^C spike-in and then subjected to m^3^C-IP-seq.

### Alignment of sequencing reads

A human transcriptome was generated based on the GRCh38 assembly of the human genome, supplemented with tRNAs and lncRNAs. Illumina sequencing reads were first trimmed to remove the first three bases from the 5′ end. The reads were then processed using fastp (version 0.23.2) [30] for adaptor removal and quality trimming. Work commands were as follows: fastp -z 9 -V -q 20 –length_required 18 -5 20 -3 20 -W 4 -M 20 -n 0 -w 8. The processed reads were mapped to reference using BWA-MEM (version 0.7.17) [31] with default parameters. After the mapping step, SAMtools (version 1.9) [32] was used to filter high-quality, uniquely aligned reads and to extract mutation information.

### Identification of m^3^C sites

The following criteria were used for identifying putative m^3^C sites. (i) Only sites in the IP group with a minimum read count of 20 were included to avoid variability in mutation detection due to insufficient reads. (ii) Sites were retained only if they were located on transcripts with abundant expression levels, defined as reads per kilobase of transcript per million mapped reads (RPKM) > 2. (iii) Only sites in the IP group with at least four mismatch counts were retained to ensure sufficient m^3^C abundance. (iv) The mismatch rate in the IP group was required to meet one of the following conditions: the mismatch rate difference between the IP and Dm (Diff) groups was at least 20%, or the Diff was at least 10% and the fold change (FC) of the mismatch rate between the IP and Dm groups was at least 2. If the mismatch rate in the Dm group was “0”, the FC was artificially set to “1000”. (v) The mismatch rate in the IP group was required to be higher than that in the Input group to distinguish sites exhibiting specific enrichment in the IP group. (vi) Only sites that met the above criteria were subjected to statistical analysis [33]. The background mutation rate was estimated using an exponential distribution model in the Dm group. To reduce variability caused by insufficient reads, only sites with at least 20 total reads were included in the background mutation rate estimation. Based on the fitted exponential distribution model, *P*-values for each site in the IP group were calculated and corrected for multiple comparisons using the false discovery rate (FDR) method. Sites with FDR values smaller than 0.01 were considered significant. (vii) Identified sites must be reproducibly detected in both replicates and not overlap with known single nucleotide polymorphisms (SNPs). Criteria (ii) and (v) were not applied to tRNAs, and criterion (ii) was not applied to lncRNAs. The sites passing these criteria are detailed in Supplementary Tables S4–S6.

### m^3^C-truncated-quantitative polymerase chain reaction (m^3^C-truncated-qPCR)

Total RNA was isolated and then reverse transcribed with the Superscript III (Invitrogen) using random primers (Takara), followed by quantitative reverse transcriptase-polymerase chain reaction. The primers used in qPCR are listed in Supplementary Table S1.

### Radioactive *in vitro* methylation assays

Genes encoding various METTL8 isoforms were cloned into the pET28a(+) vector (Addgene). The proteins were expressed in *Escherichia coli* BL21 (DE3) and purified. For the radioactive methylation assays [19], reactions were initiated by combining 1.5 μM of each METTL8 protein with 2.5 μM [^3^H]-S-adenosyladenosine (SAM; PerkinElmer) and 1 U/μl Ribolock (Thermo Fisher Scientific) in 1 × methylation buffer [50 mM Tris–HCl pH 7.0, 50 mM NaCl, 5 mM MgCl_2_, 1 mM Dithiothreitol (DTT)] at 22°C for 5 min. Following this, 1 μM synthetic oligonucleotide (Supplementary Table S1) was added, and the reaction was incubated at 22°C for 2 h. Subsequently, Proteinase K was introduced at a concentration of 1 μg/μl, and the mixture was further incubated at 22°C for 30 min. The RNA was then purified using TRIzol reagent and analyzed by scintillation counting. The purified RNA was mixed with 10 ml of scintillation liquid (Carl Roth) and measured using an LSC-8000 scintillation counter (ALOKA).

### Luciferase reporter assay

*DYNC1H1* luciferase reporters were co-transfected with pRL-TK into HEK293T cells in a 12-well plate using lipofectamine 2000 (Thermo Fisher Scientific). Cells were harvested at 24 h post-transfection. Firefly and Rinella luciferase activities were measured using the Dual-Luciferase Assay kit (Promega). The activities of the luciferase were expressed as normalized relative light units to the *DYNC1H1* luciferase reporter without m^3^C.

### Statistical analysis

Statistical analysis was performed using GraphPad Prism 9.5 software (GraphPad Software, Inc, San Diego). Data were represented as mean ± standard error of the mean (SEM) unless stated otherwise. Asterisks denoted the level of statistical significance (*P < .05, **P < .01, ***P < .001, ^****^P < .0001).

## Results

### Detection of m^3^C in mammalian mRNAs

**Figure 1.**
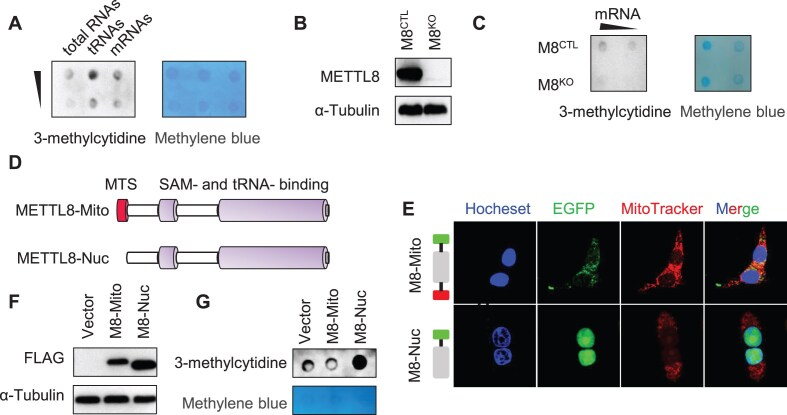
m^3^C modifications present in mRNAs. (**A**) Dot blot using anti-3-methylcytidine (m^3^C) antibody (Active Motif, left) against total RNAs, tRNAs, and mRNAs from wild-type HEK293T cells. Methylene blue staining (right) was included for loading control. (**B**) Immunoblot analysis of wild-type HEK293T cells (M8^CTL^) or *METTL8* knockout cells (M8^KO^). α-Tubulin was included for loading control. (**C**) Dot blot using anti-m^3^C antibody against mRNAs from M8^CTL^ and M8^KO^ HEK293T cells. Loading was visualized by methylene blue staining. (**D**) Schematic comparison of METTL8-Mito and METTL8-Nuc with SAM- and tRNA-binding domain, and MTS sequence indicated. (**E**) Confocal microscopy images of HEK293T cells expressing EGFP-tagged M8-Mito or M8-Nuc. The nuclei and mitochondria were stained with Hoechst and MitoTracker, respectively. Construct schematics were on the left. (**F**) Immunoblot analysis of HEK293T cells expressing FLAG-tagged M8-Mito/M8-Nuc. α-Tubulin was included for loading control. (**G**) Dot blot using anti-m^3^C antibody against mRNAs from HEK293T cells expressing FLAG-tagged M8-Mito/M8-Nuc. Loading was visualized by methylene blue staining.

The less abundance of m^3^C modification in mRNAs prevents its detection. We reasoned that antibody-based enrichment of m^3^C-containing RNAs would help resolve this question. We focused on an antibody raised against 3-methylcytidine, which was broadly used to detect m^3^C modification in DNA [34]. Since the antibody recognizes 3-methylcytidine, it might also bind the RNA form of m^3^C. Dot blot showed that the m^3^C antibody selectively bound nucleotides containing m^3^C residue and exhibited negligible binding to nucleotides containing unmodified cytosine (Supplementary Fig. S1A). To further confirm the specificity of this antibody for m^3^C, a competition assay was performed using cellular tRNAs, which are heavily modified by m^3^C. Indeed, the binding between the antibody and tRNAs could be blocked by 3-methylcytidine but not cytidine (Supplementary Fig. S1B). To explore the abundance of m^3^C in various types of RNA, we isolated mRNAs and tRNAs from total RNAs and performed dot blot analysis. Data showed that m^3^C existed in all types of RNA, with particularly high enrichment in tRNAs (Fig. 1A). To further determine whether METTL8 catalyzes m^3^C formation in mRNAs, we generated a *METTL8* knockout HEK293T cell line using the CRISPR/Cas9 genome editing system (Supplementary Fig. S1C). Knockout of *METTL8* greatly reduced m^3^C levels in mRNAs (Fig. 1B and C), supporting that METTL8 mediates m^3^C modification in mRNAs. The human *METTL8* gene has seven different protein isoforms in the NCBI database. Some isoforms contain the mitochondrial targeting signal (MTS) (hereinafter referred to as METTL8-Mito) while one METTL8 isoform misses this sequence, which is predicted to be located in the nucleus [35, 36] (hereinafter referred to as METTL8-Nuc) (Fig. 1D and Supplementary Figs S1D and E). We proposed that METTL8-Nuc is responsible for the formation of m^3^C in mRNAs. Therefore, we expressed either METTL8-Mito or METTL8-Nuc in HEK293T cells. Fluorescence examination showed that METTL8-Nuc was indeed located in the nucleus (Fig. 1E). Dot blot analysis did show that overexpression of METTL8-Nuc increased the m^3^C methylation level in mRNAs (Fig. 1F and G, and Supplementary Fig. S1F and G), without enhancing m^3^C levels in tRNAs (Supplementary Fig. S1H). We repeated these experiments in the HCT116 cell line and obtained the same results (Supplementary Fig. S2). Collectively, we provide definitive proof that METTL8-Nuc promotes m^3^C formation in mRNAs *in vivo*.

### m^3^C-IP-seq experimental design

**Figure 2.**
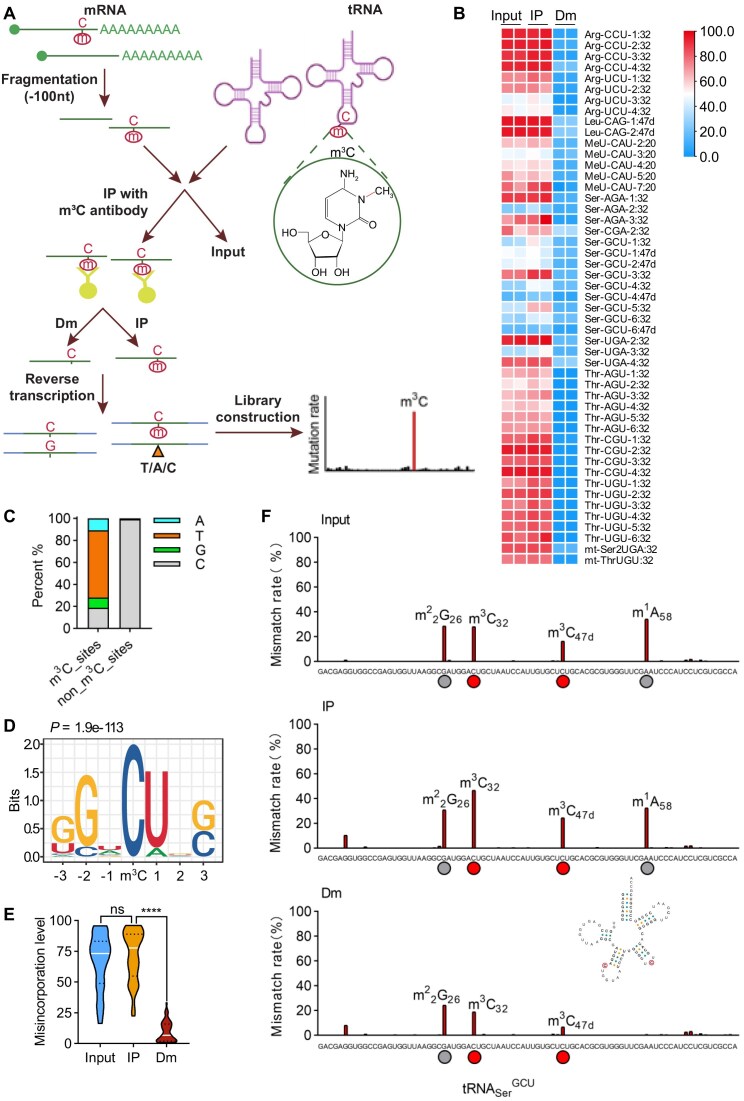
m^3^C-IP-seq captures m^3^C modification in tRNAs. (**A**) Scheme outline of m^3^C-IP-seq. (**B**) Heat map showing the mismatch rates for all m^3^C-modified tRNA sites identified from m^3^C-IP-seq datasets. Mismatch rates were shown for Input, IP, or Dm samples of two replicates. (**C**) Mutation pattern of m^3^C sites and non_m^3^C_sites in tRNAs. Shown here was the percentage of nucleotides A, C, G, and T detected at nucleotides C by following the m^3^C-IP-seq data analysis pipeline. (**D**) Consensus motif of m^3^C sites within tRNAs. (**E**) Plots depicting misincorporation levels of all tRNA m^3^C sites in different RNA samples detected by m^3^C-IP-seq. The upper and lower quartiles and the median were indicated for each group. (^****^P < .0001; two replicates, *t*-test, two-tailed). (**F**) Identification of two m^3^C sites at position 32 and 47d in cytosolic human tRN_Ser_^GCU^ by m^3^C-IP-seq. Two other modifications (at positions 26 and 58) were not sensitive to antibody enrichment or demethylation.

Previous studies have shown that m^3^C modification introduces errors during reverse transcription [14, 25, 29]. Taking advantage of this property, we developed the m^3^C-IP-seq method to map m^3^C in RNAs at single-nucleotide resolution. In this approach, RNA fragments containing m^3^C are selectively precipitated using an m^3^C-specific antibody and subsequently reverse transcribed with the high-processivity reverse transcriptase, SuperScript IV. Mutations introduced during reverse transcription are analyzed to pinpoint the precise locations of m^3^C modifications across the transcriptome. For each dataset, three types of samples were included: (i) RNA sequenced directly without immunoprecipitation (“Input”), (ii) RNA immunoprecipitated with the m^3^C antibody (“IP”), and (iii) RNA immunoprecipitated and then demethylated using the demethylase AlkB (“Dm”) [37]. This design ensures that potential false positives caused by m^3^C-independent mismatches are accounted for and minimized. To robustly identify m^3^C-specific misincorporation profiles, we developed an analytical pipeline that statistically evaluates whether misincorporation rates in the IP samples are significantly elevated. This is achieved using an exponential distribution to estimate the background mutation rate derived from the Dm group (Fig. 2A and Supplementary Fig. S3A).

### m^3^C-IP-seq captures m^3^C modification in tRNAs

To assess the sensitivity and specificity of m^3^C-IP-seq, we synthesized a 31-bp oligonucleotide with the 11th position cytidine modified with m^3^C, alongside an identical sequence without the m^3^C modification. These two oligos were mixed at varying molar ratios and used as input RNA for library preparation (Supplementary Fig. S3B). After sequencing, we evaluated the m^3^C modification levels based on mismatch rates, which varied directly and linearly with the target abundance of m^3^C, demonstrating a detection limit of 5.46% (Supplementary Fig. S3C). Despite a slight underestimation of the m^3^C level, our method exhibited robust quantitative capability for m^3^C detection. To further validate our m^3^C-IP-seq approach, we included a 10% m^3^C spike-in in one of the tRNA Input groups. The results showed an m^3^C mismatch rate of 3.21% in the Input group, 9.18% in the IP group, and 4.21% in the Dm group (Supplementary Fig. S3D). The significant mutation in the IP and Input group compared to the background mutation rate, further supports the high sensitivity and specificity of our methods in identifying m^3^C modifications.

Applying m^3^C-IP-seq to tRNAs isolated from HEK293T cells, we successfully identified 49 m^3^C modification sites corresponding to 12 cytoplasmic tRNA isoacceptors and 2 mitochondrial tRNA isoacceptors in HEK293T cells (Fig. 2B). Detailed information on the number of sites passing each filtering criterion for tRNAs is presented in Supplementary Fig. S3E. The sites where m^3^C occurred revealed strong enrichment of C→T, with less C→A or C→G transitions (Fig. 2C). This is consistent with *in vitro* data that m^3^C mainly induces A incorporation during reverse transcription [29]. Thus, the C→T transition may serve as a signature mutation for mapping m^3^C. All m^3^C sites detected by m^3^C-IP-seq and other methods including HAC-seq [28], DM-tRNA-seq [26], and HAMR [25] were summarized in Supplementary Table S3 together with documented m^3^C modification sites from the Modomics database [38]. An unbiased motif detection using MEME Suite [39] revealed that these m^3^C sites were found within a strong GGWCUNS (Fig. 2D) which is consistent with the HAC-seq identified sequence. The demethylase-mediated demethylation step significantly decreased the RT misincorporations by m^3^C modification (Fig. 2E). The effect of immunoprecipitation was not readily apparent for sites with a high mismatch rate (Supplementary Fig. S3F), but it significantly increased the sensitivity of detection for sites with a low mismatch rate (Fig. 2F). These observations suggest that m^3^C-IP-seq is sensitive and reliable in detecting adequately m^3^C at single-base resolution.

### Characterization of m^3^C-containing sites in mRNAs and lncRNAs

**Figure 3.**
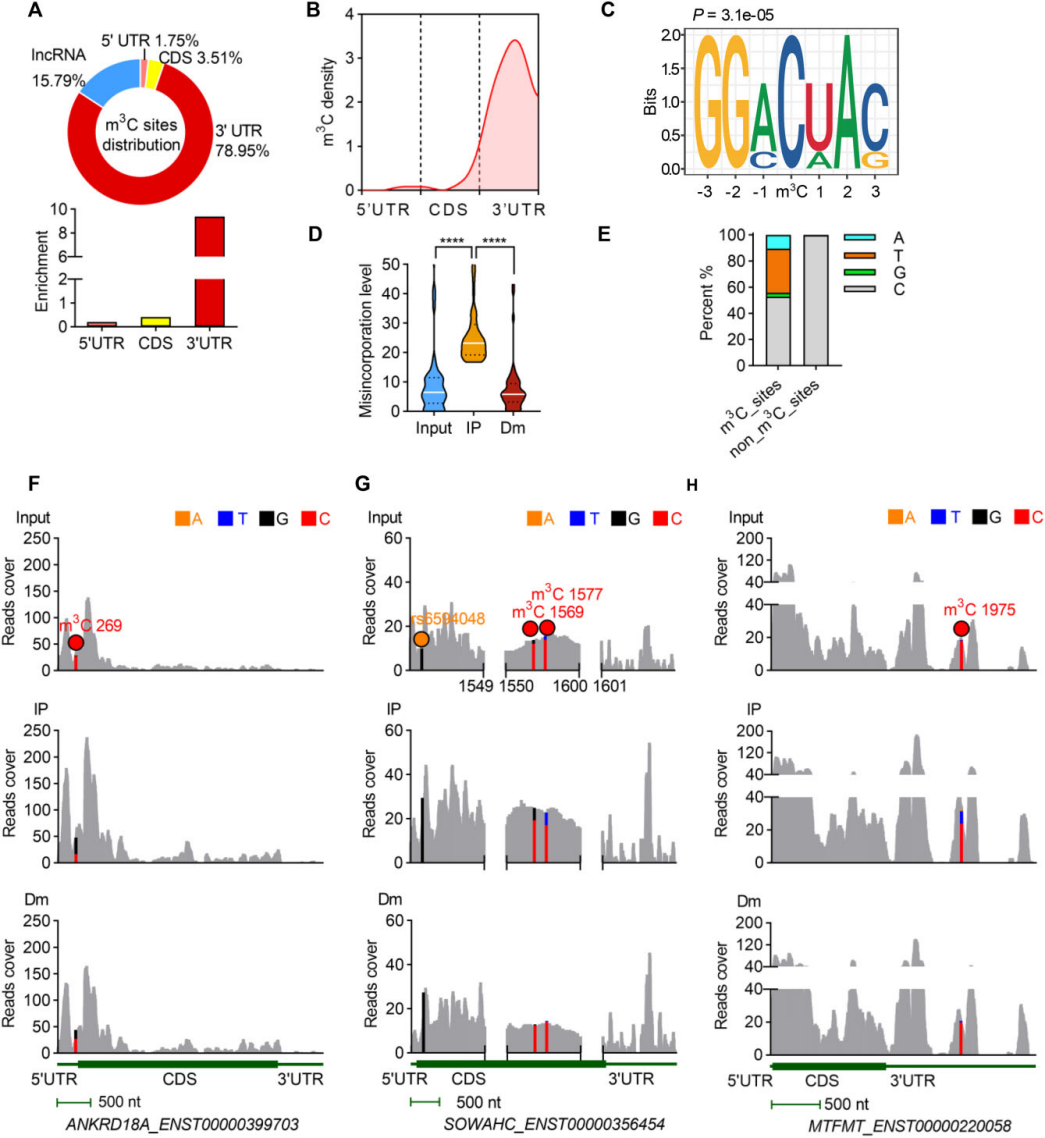
Transcriptome-wide mapping of m^3^C sites. (**A**) Pie chart showing the distribution of m^3^C sites among distinct RNA types, with a predominant enrichment in the 3′ UTR. (**B**) Metagene profiles of m^3^C sites distribution across transcripts (two replicates). (**C**) Consensus motif of m^3^C sites within mRNAs. (**D**) Plots depicting misincorporation levels of all mRNA m^3^C sites in different RNA samples detected by m^3^C-IP-seq. The upper and lower quartiles and the median were indicated for each group. (^****^P < .0001; two replicates, *t*-test, two-tailed). (**E**) Mutation pattern of m^3^C sites and non_m^3^C_sites in mRNAs. Shown here was the percentage of nucleotides A, C, G, and T detected at nucleotides C by following the m^3^C-IP-seq data analysis pipeline. (**F–** **H**) Representative views of the identified m^3^C site in the 5′ UTR of *ANKRD18A* (**F**), the CDS of *SOWAHC* (**G**), and the 3′ UTR of *MTFMT* (**H**). rs6594048 indicates an immunoprecipitation/demethylation-insensitive signal corresponding to an annotated SNP. For marked positions, bar plots represent the base composition, while unmarked regions display the measured counts. The scale bars are shown at the bottom of each panel.

Having validated the ability of m^3^C-IP-seq to detect m^3^C in tRNAs, we then sought to detect transcriptome-wide m^3^C methylome. Collectively, we identified 48 high-confidence putative m^3^C sites in mRNAs (Supplementary Fig. S4B) and 9 sites in lncRNAs (Supplementary Fig. S4C). Detailed information on the number of sites passing each filtering criterion for mRNAs and lncRNAs is presented in Supplementary Fig. S5. Notably, the majority of m^3^C sites in mRNAs were enriched within the 3′ UTR region (Fig. 3A and B, and Supplementary Fig. S4A) and exhibited a conserved sequence motif, GGACUAC (Fig. 3C). Similarly, the motif GGACUUC was predominant among lncRNA-associated m^3^C sites (Supplementary Fig. S4D). Interestingly, the sequence motifs associated with m^3^C in both mRNAs and lncRNAs closely resembled the motif observed in tRNAs (Fig. 2D). Analysis of misincorporation rates revealed that most m^3^C sites in mRNAs exhibited very low misincorporation rates (below 10%) in the absence of antibody-mediated enrichment, suggesting that these modifications occur at low stoichiometries (Fig. 3D). We systematically evaluated the mutation pattern of the identified m^3^C sites in mRNAs, C→T transitions during reverse transcription were also the most prevalent (Fig. 3E). Representative examples of m^3^C sites identified in different mRNA regions are shown in Fig. 3F-H. An additional site of high mutation, insensitive to the immunoprecipitation and demethylase treatment, appeared in *SOWAHC* mRNAs (Fig. 3G). By referring to the SNP database [40], we found that this position belongs to an annotated SNP site, demonstrating the robustness of our approach in distinguishing true m^3^C sites from false signals (SNP, etc.).

### METTL8 catalyzes m^3^C modifications in mRNAs and lncRNAs

**Figure 4.**
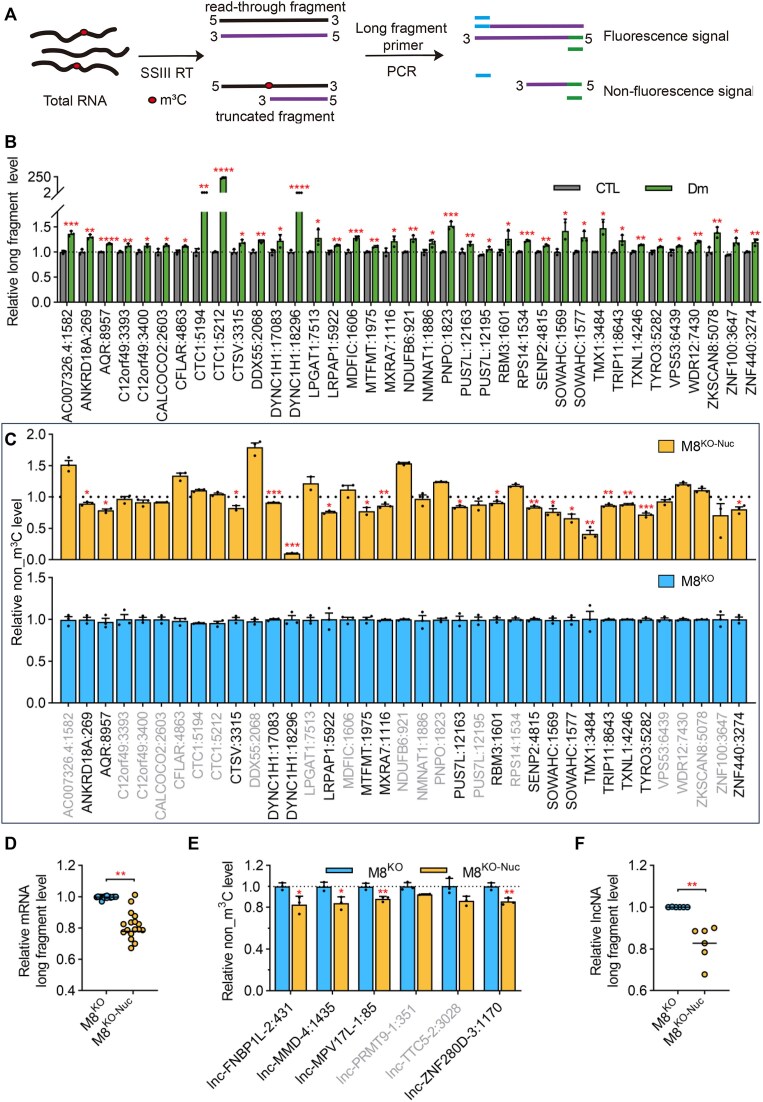
METTL8-Nuc catalyzes m^3^C methylation in mRNAs and lncRNAs. (**A**) Schematic diagram showing the design of m^3^C modification detection by qPCR. (**B**) Relative long fragment expressions of mRNA m^3^C sites with or without demethylation detected by m^3^C-truncated-qPCR. Data were normalized to the value of M8^CTL^. Values represent mean ± SEM (*P < .05, **P < .01, ***P < .001, ^****^P < .0001; three independent experiments, *t*-test, two-tailed). Successfully validated sites were highlighted. (**C**) qPCR measurement of relative non_m^3^C level (m^3^C_fragment/non_m^3^C_fragment ratio) for mRNAs identified to contain m^3^C modification in HEK293T cells. Data were normalized to the value of M8^KO^. Values represent mean ± SEM (*P < .05, **P < .01, ***P < .001, ^****^P < .0001; 3 independent experiments, *t*-test, two-tailed). Successfully validated sites were highlighted. (**D**) Relative expression of long fragments for mRNA m^3^C sites in different RNA samples detected by m^3^C-truncated-qPCR. The median was indicated for each group. (**P < .01; *t*-test, two-tailed). (**E**) qPCR measurement of the relative non_m^3^C level for lncRNAs identified to contain m^3^C modification in HEK293T cells. Data were normalized to the value of M8^CTL^. Values represent mean ± SEM. The median was indicated for each group. (*P < .05, **P < .01; 3 independent experiments, *t*-test, two-tailed). (**F**) Relative expression of long fragments for lncRNA m^3^C sites in different RNA samples detected by m^3^C-truncated-qPCR. The median was indicated for each group. (**P < .01; *t*-test, two-tailed).

To identify mRNAs methylated by METTL8-Nuc, we performed an independent m^3^C-truncated-qPCR assay to validate the presence of m^3^C sites identified by m^3^C-IP-seq. This assay utilizes the reverse transcription blockade caused by m^3^C modification when using SuperScript III reverse transcriptase (SS III). Consequently, only RNAs without m^3^C modifications generate long cDNA fragments (Fig. 4A). The expression change of long fragments across different groups reflects alterations in the m^3^C modification level, and the ratio of m^3^C_fragment/non_m^3^C_fragment indicates the level of non_m^3^C modification levels. Using this approach, we validated 37 out of 48 sites in mRNAs capable of inducing truncation (Fig. 4B and Supplementary Fig. S6A). Among these, 18 m^3^C sites were confirmed to be catalyzed by METTL8-Nuc upon reintroducing METTL8-Nuc into METTL8 knockout cells (Fig. 4C). Importantly, these reductions were independent of changes in RNA expression levels (Fig. 4D). Beyond mRNAs, we also validated nine m^3^C sites in lncRNAs identified by m^3^C-IP-seq, successfully confirming four of these sites as METTL8-Nuc-dependent (Supplementary Fig. S6B and Fig. 4E and F). These results suggest that, similar to mRNAs, m^3^C modifications in lncRNAs are dynamically regulated by METTL8-Nuc. To further explore METTL8’s catalytic activity, we conducted *in vitro* methylation assays using [^3^H]-SAM as the methyl donor and purified METTL8 protein variants: METTL8-Mito, METTL8-Nuc, and a truncated form lacking the methyltransferase domain (METTL8-ΔSAM). Since the m^3^C modification motifs in mRNAs and lncRNAs closely resemble that in tRNAs, we utilized this conserved motif to evaluate METTL8-Nuc’s catalytic activity. The results revealed that METTL8-Nuc exhibited catalytic activity toward this motif, achieving approximately 30% of the activity observed for METTL8-Mito (Supplementary Fig. S6C). These findings confirm the authenticity of the identified m^3^C sites in mRNAs and highlight the evolutionary conservation of the m^3^C modification motif across different RNA types.

### METTL8-mediated m^3^C methylation contributes to *DYNC1H1* mRNA decay

**Figure 5.**
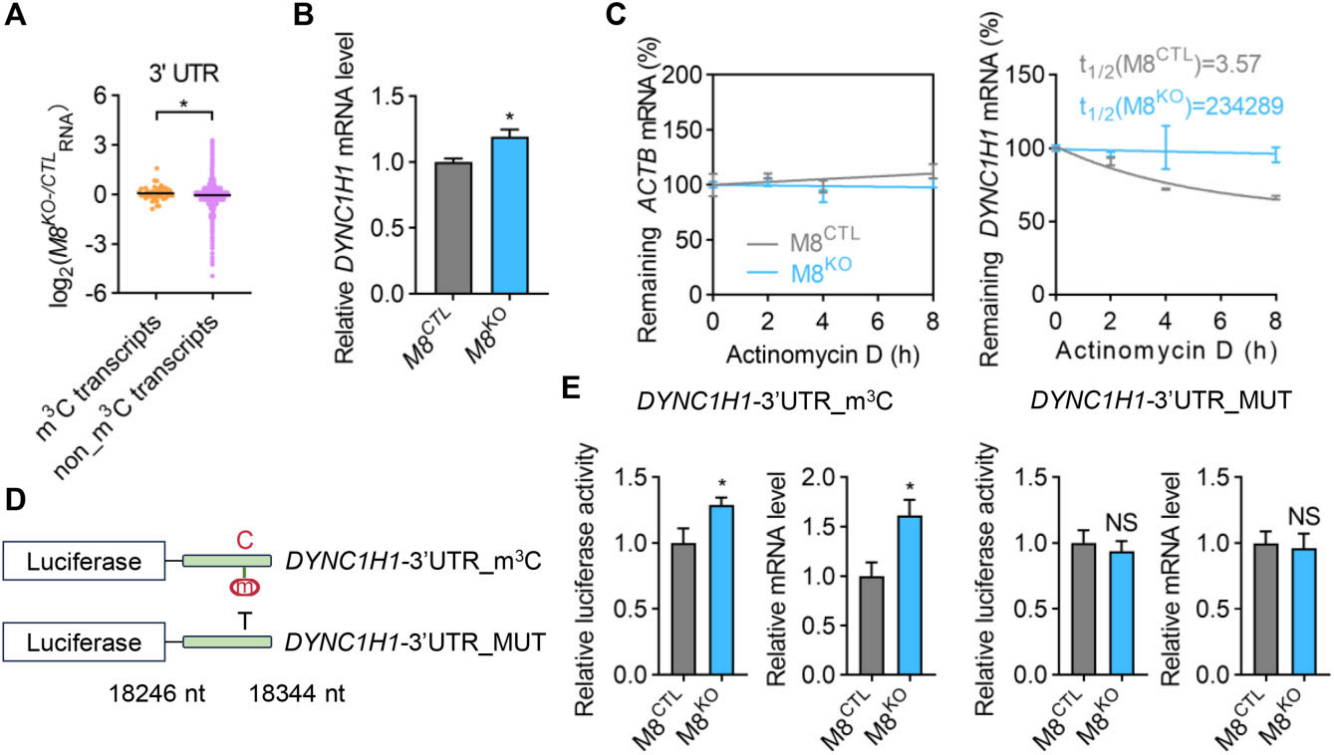
m^3^C methylation contributes to *DYNC1H1* mRNA decay. (**A**) Violin plots showing cumulative distribution log2-fold changes in mRNA expression between M8^CTL^ and M8^KO^ HEK293T cells for m^3^C transcripts and non_m^3^C transcripts. The median was indicated for each group (*P < .05; unpaired *t*-test with Welch’s correction). (**B**) The mRNA level of *DYNC1H1* in wild-type HEK293T cells (M8^CTL^) and *METTL8* knockout cells (M8^KO^). Data were normalized to the value of M8^CTL^. Values represent mean ± SEM. The median was indicated for each group (*P < .05; 3 independent experiments, *t*-test, two-tailed). (**C**) The remaining mRNA levels of *ACTB* (β-actin) and *DYNC1H1* after ActD treatment in wild-type and *METTL8*-knockout HEK293T cells. The half-life (t_1/2_) of *DYNC1H1* mRNA is indicated for both groups. Error bars represent the mean ± standard deviation (SD) of three independent experiments. (**D**) Schematic of luciferase constructs with the predicted m^3^C sites and MUT sites in the 3′ UTR of *DYNC1H1* mRNA. (**E**) Luciferase activity and mRNA level of the constructs as indicated in panel (D) in HEK293T cells (*P < .05, 3 independent experiments, *t*-test, two-tailed).

Given that m^3^C sites are primarily located in the 3′ UTR regions of mRNAs, we hypothesized that m^3^C modifications regulate mRNA metabolism [41–43]. Thus, we conducted RNA-seq and compared the expression levels of transcripts with or without m^3^C. We observed a significant increase in the abundance of m^3^C-modified transcripts within the 3′ UTR following *METTL8* knockout compared to non-m^3^C transcripts (Fig. 5A), supporting a central role for m^3^C in promoting mRNA decay. To further validate this finding, we focused on *DYNC1H1* mRNA (ENST00000360184), which was confirmed to contain m^3^C in its 3′ UTR. After *METTL8* knockout, *DYNC1H1* mRNA levels increased (Fig. 5B). We then assessed the decay kinetics of *DYNC1H1* mRNA by blocking *de novo* RNA synthesis using actinomycin D (ActD). Our results revealed that the half-life of *DYNC1H1* mRNA was significantly extended in *METTL8* knockout cells compared to wild-type HEK293T cells (Fig. 5C). To explore whether the m^3^C modification in the 3′ UTR directly mediates *DYNC1H1* mRNA decay, we cloned the m^3^C peak (18246−18344 nt of the transcript ENST00000360184) into a luciferase reporter and generated a C-to-T mutation at the m^3^C site as a control (Fig. 5D). METTL8 knockout increased luciferase activity in the *DYNC1H1*-3′UTR_m^3^C construct but not in the C-to-T mutant construct (Fig. 5E). Collectively, these results indicated that the m^3^C modification in 3′ UTR of *DYNC1H1* promotes its mRNA decay.

### m^3^C methylation is dynamically regulated by hypoxia

**Figure 6.**
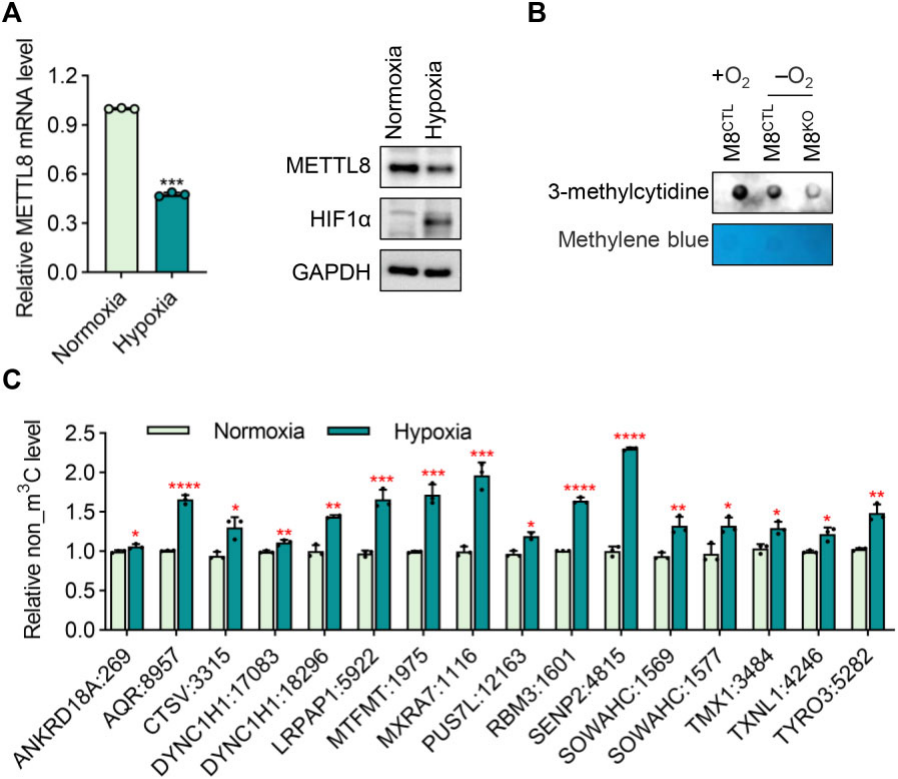
m^3^C methylation is dynamically regulated by hypoxia. (**A**) The mRNA and protein levels of *METTL8* in HEK293T cells treated under normoxia or hypoxia conditions. (**B**) Dot blot analysis of m^3^C level of mRNAs extracted from wild-type and *METTL8* knockout HEK293T cells treated under normoxia or hypoxia conditions. Loading was visualized by methylene blue staining. (**C**) Relative non_m^3^C level (m^3^C_fragment/non_m^3^C_fragment ratio) for mRNAs identified to contain m^3^C modification in HEK293T cells treated under normoxia or hypoxia conditions. Data were presented as mean ± SEM (*P < .05, **P < .01, ***P < .001,^****^P < .0001; three independent experiments, *t*-test, two-tailed).

We investigated the dynamic alteration of m^3^C under different conditions. We observed a reduction in *METTL8* expression in HEK293T cells under hypoxia conditions for 12 h (Fig. 6A). As anticipated, the hypoxia-induced decrease in METTL8 levels led to a progressive reduction in m^3^C methylation in mRNAs (Fig. 6B). m^3^C-truncated-qPCR experiments demonstrated the reductions in m^3^C modification in 16 mRNAs which have been validated to contain m^3^C modifications (Fig. 6C). These data indicate that m^3^C methylation in mRNAs is dynamically regulated by hypoxia.

## Discussion

One major factor that limits understanding the role of m^3^C in mRNA metabolism is the unknown transcriptomic distribution of this modification. Our m^3^C-IP-seq provides a valuable tool to bridge this gap and allows global mapping of the methylome to uncover its fundamental properties. m^3^C-IP-seq includes four key steps: (i) the m^3^C antibody to enrich m^3^C-containing mRNAs, (ii) m^3^C-induced misincorporation in cDNA synthesis, (iii) an *in vitro* demethylation step, which enables us to distinguish true m^3^C sites from false signals (SNP, other modifications, etc.) in the transcriptome; and (iv) the incorporation of statistical testing to identify m^3^C sites with significantly elevated mismatch rates in the IP group compared to the background mutation rate derived from the Dm group, with FDR < 0.01. By a combination of these four steps, m^3^C-IP-seq permits mapping m^3^C modification with high sensitivity and confidence.

Several techniques, such as ARM-seq [24], HAMR [25], DM-tRNA-seq [26], AlkAniline-seq [27], and HAC-seq [28] have been developed for mapping m^3^C RNA modification. ARM-seq and DM-tRNA-seq identify m^1^A, m^3^C, and m^1^G modifications, and HAMR detects m^1^A, m^2^G, and m^3^C modifications. All the three methods leverage these modifications' ability to induce termination or misincorporation during reverse transcription. AlkAniline-seq locates m^7^G and m^3^C modification sites by exposing RNA to alkaline conditions followed by aniline cleavage. HAC-seq specifically targets m^3^C modification, using hydrazine and aniline treatment to cleave at the modification sites. While these techniques effectively map m^3^C in tRNAs, they struggle to efficiently detect less abundant m^3^C modifications, particularly within mRNAs. In contrast, our m^3^C-IP-seq enhances specificity and sensitivity by using the m^3^C antibody to enrich m^3^C-containing RNA fragments. This approach holds promise for advancing our understanding of m^3^C-regulated mRNA metabolism.

It should be noted that METTL8 was recently identified as a mitochondrial protein that catalyzed m^3^C_32_ methylation of mitochondrial tRNAs. The MTS resides at the very N terminus of METTL8 [16, 17]. However, by searching the NCBI database, we found that several isoforms of METTL8 lack the MTS, which might preclude their mitochondria localization. Our immunofluorescence experiments revealed that METTL8 lacking MTS was localized in the nucleus in HEK293T and HCT116 cells (Fig. 1E and Supplementary Fig. S2E), offering the possibility that METTL8 could catalyze m^3^C formation in both the nucleus and mitochondria. Our findings demonstrated that m^3^C is distributed across the human transcriptome, with a marked enrichment in the 3′ UTR region in mRNAs. The m^3^C sites in mRNAs are located within the GGACUAC motif, similar to the m^3^C motif found in tRNAs. Moreover, we observed that m^3^C is dynamically regulated by hypoxia, where hypoxia reduces METTL8 expression and, consequently, m^3^C methylation in mRNAs. This stress-induced epitranscriptomic modulation highlights the functional significance of m^3^C regulation in response to physiological changes.

Previous studies have underscored the pivotal roles of RNA modifications in cellular processes, particularly emphasizing their impact on mRNA stability [42, 44, 45], translation efficiency [46, 47], and interactions with RNA-binding proteins [48]. Consistent with these concepts, we showed that m^3^C modification in the 3′ UTR contributes to the degradation of m^3^C-modified mRNAs, linking this epitranscriptomic mark to mRNA turnover. However, the low abundance of m^3^C and its role in promoting transcript degradation make the identification of m^3^C sites on low-expression transcripts particularly challenging, requiring high sequencing depth. While our approach primarily focuses on high-expression transcripts, we cannot exclude the possibility of bona fide m^3^C modifications in low-expression transcripts.

The subcellular localization of METTL8 is crucial for determining its substrate specificity. *In vivo* experiments showed that knockout of *METTL8* significantly reduced m^3^C levels in mRNAs (Fig. 1C and Supplementary Fig. S2D). METTL8-Nuc specifically enhanced m^3^C levels in mRNAs (Fig. 1G and Supplementary Fig. S2G), without enhancing m^3^C levels in tRNAs (Supplementary Fig. S1H). *In vitro* methylation assays also demonstrated that METTL8-Mito exhibited robust catalytic activity towards the conserved motif observed in tRNAs and mRNAs, whereas METTL8-Nuc showed relatively low catalytic activity towards it (Supplementary Fig. S6C). The low catalytic activity of METTL8-Nuc on mRNAs may be influenced by various factors. First, other modifications in the sequence may affect the catalytic activity. For instance, t^6^A in the sequence has been shown to enhance METTL8-mediated m^3^C_32_ methylation of mitochondrial tRNA_Thr/Ser_^UCN^ [19]. Second, cofactors could further modulate METTL8-Nuc’s catalytic activity in mRNAs, exemplified by the involvement of *Hs*SARS1 in enhancing METTL6-mediated tRNA_Ser_^GCU^ C_32_ methylation [49]. Addressing these questions will be a focus of future studies.

Collectively, the m^3^C-IP-seq allows global mapping of m^3^C at single nucleotide resolution, which could be widely used to detect m^3^C not only in tRNAs but also in mRNAs and lncRNAs. The m^3^C map generated in human cell lines provides a reference and resource for future investigations to elucidate the biological function and mechanisms of this epitranscriptomic mark.

## Supplementary Material

gkaf153_Supplemental_Files

## Data Availability

Sequencing data have been deposited into the Gene Expression Omnibus (GEO) under the accession number GEO: GSE287299 (https://www.ncbi.nlm.nih.gov/geo/query/acc.cgi?acc=GSE287299).
