# Supplementary material for: Transcriptome-wide mapping of N3-methylcytidine modification at single-base resolution
Source: Nucleic Acids Res. 2025 Mar 12;53(5):gkaf153. doi: 10.1093/nar/gkaf153 (PMC11897884; doi:10.1093/nar/gkaf153)
Supplement: gkaf153_Supplemental_Files [file gkaf153_supplemental_files.zip › Supplementary_Information.docx]

**Supplementary Information**

**Transcriptome-wide mapping of N^3^-methylcytidine modification at single-base resolution**

Yunyi Gao^1,#^, Jingyu Hou^1,2,#^, Saisai Wei^3,#^, Canlan Wu^1^, Sujun Yan^1^, Jia Sheng^4^, Jun Zhang^1,*^, Zhanghui Chen^2,*^, Xiangwei Gao^1,*^.

^1^ Department of Clinical Laboratory of Sir Run-Run Shaw Hospital, and School of Public Health, Zhejiang University School of Medicine, Hangzhou 310058, China.

^2^ Zhanjiang Institute of Clinical Medicine, Zhanjiang Central Hospital, Zhanjiang 524000, China.

^3^ Key Laboratory of Laparoscopic Technology of Zhejiang Province, Department of General Surgery, Sir Run-Run Shaw Hospital, Zhejiang University School of Medicine, Hangzhou 310016, China.

^4^ Department of Chemistry, The RNA Institute, University at Albany SUNY, NY 12222, USA.

^#^ These authors contributed equally.

^*^ Correspondence should be addressed to Xiangwei Gao: [xiangweigao@zju.edu.cn](mailto:xiangweigao@zju.edu.cn); or Zhanghui Chen: [zjcell@126.com](mailto:zjcell@126.com); or Jun Zhang: [jameszhang2000@zju.edu.cn](mailto:jameszhang2000@zju.edu.cn).

**Supplementary Results**

**Supplementary Table 1**

Sequences of oligos were used in this study.

| Oligos of cDNA cloning for plasmids construction | |
| --- | --- |
| hM8-Mito_cDNA F | 5'-ACCTCCATAGAAGATTCTAGAGCCACCATGAATATGATTTGG-3' |
| hM8-Nuc_cDNA F | 5'-ACCTCCATAGAAGATTCTAGAGCCACCATGCAGTGGTCTAAG-3' |
| hM8_cDNA-FLAG R | 5'-GTCATCCTTGTAATCGGATCCGTCTTGTGAAAGGAG-3' |
| hM8_cDNA-EGFP R | 5'-gcccttgctcaccatGGATCCGTCTTGTGAAAGGAG-3' |
| Oligos of CRISPR/Cas9 | |
| h*METTL8*_gRNA F | 5'-CACCGAGGATCGTAATTGGCTGTTG-3' |
| h*METTL8*_gRNA R | 5'-AAACCAACAGCCAATTACGATCCTC-3' |
| Oligos of m^3^C-truncated-qPCR used to validate m^3^C sites | |
| 3' adaptor | 5'-rApp-TGGAATTCTCGGGTGCCAAGG-NH_2_-3' |
| RT primer | 5'-GCCTTGGCACCCGAGAATTCCA-3' |
| 5' DNA adaptor | 5'-Phos-GATCGTCGGACTGTAGAACTCTGAAC-NH_2_-3' |
| Universal adaptor | 5'-AATGATACGGCGACCACCGAGATCTACACGTTCAGAGTTCTACAGTCCGA-3' |
| Index 1 | 5'-CAAGCAGAAGACGGCATACGAGATCGTGATGTGACTGGAGTTCCTTGGCACCCGAGAATTCCA-3' |
| Index 2 | 5'-CAAGCAGAAGACGGCATACGAGATACATCGGTGACTGGAGTTCCTTGGCACCCGAGAATTCCA-3' |
| Index 3 | 5'-CAAGCAGAAGACGGCATACGAGATGCCTAAGTGACTGGAGTTCCTTGGCACCCGAGAATTCCA-3' |
| Index 4 | 5'-CAAGCAGAAGACGGCATACGAGATTGGTCAGTGACTGGAGTTCCTTGGCACCCGAGAATTCCA-3' |
| Index 5 | 5'-CAAGCAGAAGACGGCATACGAGATCACTGTGTGACTGGAGTTCCTTGGCACCCGAGAATTCCA-3' |
| Index 6 | 5'-CAAGCAGAAGACGGCATACGAGATATTGGCGTGACTGGAGTTCCTTGGCACCCGAGAATTCCA-3' |
| m^3^C RNA oligo | 5'-UAGUCUGCACm^3^CUGCACCAGUCGCUCAGGGAU-3' |
| non_m^3^C RNA oligo | 5'-UAGUCUGCACCUGCACCAGUCGCUCAGGGAU-3' |
| Oligos of m^3^C-truncated-qPCR used to validate m^3^C sites | |
| h-ACTB F | 5'-CACCATTGGCAATGAGCGGTTC-3' |
| h-ACTB R | 5'-AGGTCTTTGCGGATGTCCACGT-3' |
| h-GAPDH F | 5'-ATCACTGCCACCCAGAAGAC-3' |
| h-GAPDH R | 5'-TTTCTAGACGGCAGGTCAGG-3' |
| AC007326.4_m^3^C F | 5'-CACTTGTGGGTGTTTCTCGA-3' |
| AC007326.4_m^3^C R | 5'-CTTGTGACCCTGACACATCC-3' |
| AC007326.4_non_m^3^C F | 5'-GAAGCACAGGCAGGTTATGG-3' |
| AC007326.4_non_m^3^C R | 5'-TCACCGAAAAGAGCCTGTAC-3' |
| ANKRD18A_m^3^C F | 5'-CCCACCACCCGCTCCTGAG-3' |
| ANKRD18A_m^3^C R | 5'-GGGGTGGAAAGGCCACGAG-3' |
| ANKRD18A_non_m^3^C F | 5'-CAAGGTAACTCTGGTTCTGGC-3' |
| ANKRD18A_non_m^3^C R | 5'-GGAGAAACAGCGGATGAAATATTTTC-3' |
| AQR_m^3^C F1 | 5'-ACCTCAGGTGATCCGCCCGC-3' |
| AQR_m^3^C R1 | 5'-GCCTGTAATCCCAGCACTTTGGG-3' |
| AQR_m^3^C F2 | 5'-TCTCGATCTCCTGACCTCAGGTGATC-3' |
| AQR_m^3^C R2 | 5'-AGCACTTTGGGAGGCCCAGG-3' |
| AQR_non_m^3^C F | 5'-GTCTCTTCCGGGGCAGATG-3' |
| AQR_non_m^3^C R | 5'-TAGGAGCTGTATCTGCACCG-3' |
| C12orf49_m^3^C F1 | 5'-GCCTCAGCCTTCCAAAGTGC-3' |
| C12orf49_m^3^C R1 | 5'-TGGGGCGTGGTGGCTTATG-3' |
| C12orf49_m^3^C F2 | 5'-ATCCACCTGCCTCAGCCTTC-3' |
| C12orf49_m^3^C R2 | 5'-GGCTTATGCCTGTAATCCCAG-3' |
| C12orf49_non_m^3^C F | 5'-GCAAACGTAGCCGAGTTCATCC-3' |
| C12orf49_non_m^3^C R | 5'-CCATCCCGTGGAAAGTGCAG-3' |
| CALCOCO2_m^3^C F | 5'-GGGAGGCTAAGGCATGAGAATC-3' |
| CALCOCO2_m^3^C R | 5'-CCTCTGCCTCCCAGGTTCAA-3' |
| CALCOCO2_non_m^3^C F | 5'-AGAGAGAGTGGCAGAACACG-3' |
| CALCOCO2_non_m^3^C R | 5'-ATTGTCCAATTTGTGACAAGATCTTC-3' |
| CFLAR_m^3^C F | 5'-TCTCCCCACGGTCTCCCTCT-3' |
| CFLAR_m^3^C R | 5'-TACAGTCCAGCTTCGGCTCG-3' |
| CFLAR_non_m^3^C F | 5'-GCACTGCAGGTACAGGGATG-3' |
| CFLAR_non_m^3^C R | 5'-AGTTCACCGAGAAGCTGACTTC-3' |
| CTC1_m^3^C F1 | 5'-TATGACGTAGTCGGCAGGATTC-3' |
| CTC1_m^3^C R1 | 5'-TTAAGGCGATGGACTAGAAATCCAT-3' |
| CTC1_m^3^C F2 | 5'-CGGGGAGACCCCAATGGATTTC-3' |
| CTC1_m^3^C R2 | 5'-TGGCCGAGTGGTTAAGGCGA-3' |
| CTC1_non_m^3^C F | 5'-GCTCCAGTTCCAGCTCCTG-3' |
| CTC1_non_m^3^C R | 5'-ACAGAACTGCGAGTGTCCAAG-3' |
| CTSV_m^3^C F | 5'-CGTTCCTAAACTACAAACAATAGC-3' |
| CTSV_m^3^C R | 5'-CATGTCCTTAAGGCACAGAT-3' |
| CTSV_non_m^3^C F | 5'-ACAGTGGTTGTTCTTGTCTTTG-3' |
| CTSV_non_m^3^C R | 5'-CAAGTATTGGCTCGTCAAAAACAGC-3' |
| DDX55_m^3^C F | 5'-CCTCCCAAAGTGCTGGGATTATAGGCA-3' |
| DDX55_m^3^C R | 5'-TCCAGTCTTGGCCGGGTGCG-3' |
| DDX55_non_m^3^C F | 5'-TCATCTTCCAAATCTGAGATCC-3' |
| DDX55_non_m^3^C R | 5'-GACTCTTGAAAAAACTTAAGAAAGG-3' |
| DYNC1H1_m^3^C F1 | 5'-CTTGGTGGTGGGCGCCTGTA-3' |
| DYNC1H1_m^3^C R1 | 5'-TGCCTCAGCCTCCCAAGTAG-3' |
| DYNC1H1_m^3^C F2 | 5’-CAAGGTGGGCAGATCACCTG-3’ |
| DYNC1H1_m^3^C R2 | 5’-AGGCTGGTCTCGAACTCCTG-3’ |
| DYNC1H1_non_m^3^C F | 5'-GGTGTTTGTCTGCTTGACCCAG-3' |
| DYNC1H1_non_m^3^C R | 5'-CCAATGCCATCTCAACCGCC-3' |
| FBXL20_m^3^C F | 5'-GATAGAAACTGGCCAGGTGC-3' |
| FBXL20_m^3^C R | 5'-TCCCAAAGTGCTGGGATTAC-3' |
| FBXL20_non_m^3^C F | 5'-CTGGAGCACTTGAAGAGCTG-3' |
| FBXL20_non_m^3^C R | 5'-GTGGACTTTAATATTGGGTAAATGG-3' |
| GEMIN6_m^3^C F | 5'-CTGCACTCCAGCCTGAGTGA-3' |
| GEMIN6_m^3^C R | 5'-TGTTTGTTTTTGAGACAGAGTCTTGC-3' |
| GEMIN6_non_m^3^C F | 5’-AGTCAACACCCAAACTGGCA -3’ |
| GEMIN6_non_m^3^C R | 5'-GTAGTTAAAACCCATCCTTTATACTCATTC-3' |
| ING5_m^3^C F1 | 5'-TTAGCTGGGTGGCCAGGCG-3' |
| ING5_m^3^C R1 | 5'-GTGCTGGGATTACAGGCGTG-3' |
| ING5_m^3^C F2 | 5'-GTCTGGCCAATATGGTGAAACCC-3' |
| ING5_m^3^C R2 | 5'-GGATTACAGGCGTGAGCCAC-3' |
| ING5_non_m^3^C F | 5'-CCTACTTCTTCTTCCTCTTTTCC-3' |
| ING5_non_m^3^C R | 5'-CTTACCACGAAACCCAAAGG-3' |
| LPGAT1_m^3^C F | 5'-CTGGTCAGCAATAGATCTCTG-3' |
| LPGAT1_m^3^C R | 5'-AGACAAGGAACAGACTGGGG-3' |
| LPGAT1_non_m^3^C F | 5'-TCCACGTCAATTCCTAAAACAGG-3' |
| LPGAT1_non_m^3^C R | 5'-CCATAAGGAAGCTGTTTCCAG-3' |
| LRPAP1_m^3^C F | 5'-CCTGCCTCAGCCTCCAGAGT-3' |
| LRPAP1_m^3^C R | 5'-GTGGTGCATGCCTGTAATCC-3' |
| LRPAP1_non_m^3^C F | 5'-GAGGAGCTCAAGCACTTCG-3' |
| LRPAP1_non_m^3^C R | 5'-TCTCTGCGTGCCTCAGCTTC-3' |
| MDFIC_m^3^C F | 5'-GCTGGTCTCGAACTCCTGAG-3' |
| MDFIC_m^3^C R | 5'-CATTTTGGGAGGCCAAGGTG-3' |
| MDFIC_non_m^3^C F | 5'-ATGATTCACAACAGGCATCC-3' |
| MDFIC_non_m^3^C R | 5'-TCATGTGGCATCTGCACCTC-3' |
| MTFMT_m^3^C F | 5'-CATGGTGGCGGACGCCTGTA-3' |
| MTFMT_m^3^C R | 5'-TGCCTCAGCCTCCCAAGTAG-3' |
| MTFMT_non_m^3^C F | 5'-TCTACAATGGATATTTGCACC-3' |
| MTFMT_non_m^3^C R | 5'-GTTGGAAGTCTGAGAGTCTG-3' |
| MXRA7_m^3^C F | 5'-ATCACCTGAGGTCAGGAGTTCG-3' |
| MXRA7_m^3^C R | 5'-TTTTGCCATGCTGGCCAGGC-3' |
| MXRA7_non_m^3^C F | 5'-ATGTCGTAGAGCCGGAGCTG-3' |
| MXRA7_non_m^3^C R | 5'-GCTGCCATCTTCAAGCTCATGAAAG-3' |
| NDUFB6_m^3^C F | 5'-GATCTCCTGACTTCGTGATCTGC-3' |
| NDUFB6_m^3^C R | 5'-ATCCCAGCACTTTGGGAGGC-3' |
| NDUFB6_non_m^3^C F | 5'-GTTGATCAGGAAATTCTTTCATTGG-3' |
| NDUFB6_non_m^3^C R | 5'-CTGAAAAACCATATGGCATAGTTG-3' |
| NMNAT1_m^3^C F | 5'-AGCTGTCAGGCTTTTTTTTTTTTTTGAGAT-3' |
| NMNAT1_m^3^C R | 5'-CAGCCTGGGTGACAGAGCG-3' |
| NMNAT1_non_m^3^C F | 5'-CAGGATGACCCCAGCATTCC-3' |
| NMNAT1_non_m^3^C R | 5'-GCATTCGCTACTTGGTACCAG-3' |
| PABPN1_m^3^C F | 5'-CCAGGCAATCTGGAGGGGCAG-3' |
| PABPN1_m^3^C R | 5'-TACCACCCACCCCACCCCTC-3' |
| PABPN1_non_m^3^C F | 5'-GGAGCAGCCCATCTATCCTG-3' |
| PABPN1_non_m^3^C R | 5'-TTTTAACAGCAGGCCCCGGGGTC-3' |
| PNPO_m^3^C F | 5'-TGTGGTGGCGTGTGCCTGT-3' |
| PNPO_m^3^C R | 5'-CTGCCTCAGCCTCCCAAGTAG-3' |
| PNPO_non_m^3^C F | 5'-GCCTTCCACACGCACCTGAC-3' |
| PNPO_non_m^3^C R | 5'-GAAAAGAGCTGGACTCTAATCCCTTTG-3' |
| PUS7L_m^3^C F1 | 5'-TTGAACCCAAGAGGCAGAGGT-3' |
| PUS7L_m^3^C R1 | 5'-GTGCAGTGGCACGATCTTGG-3' |
| PUS7L_m^3^C F2 | 5'-TAGCTCCTTGGGAGGCTGAT-3' |
| PUS7L_m^3^C R2 | 5'-TGCCTCTTGGGTTCAAGTGATT-3' |
| PUS7L_non_m^3^C F | 5'-TGTCTTCATCCAAACAGACCAAATC-3' |
| PUS7L_non_m^3^C R | 5'-TGCGCATATTCTATGTTCACGC-3' |
| RBM3_m^3^C F1 | 5'-AGCAATTCTCCTGCCTCAGC-3' |
| RBM3_m^3^C R1 | 5'-CGGGTGCATGTAATCCCAGCTA-3' |
| RBM3_m^3^C F2 | 5'-TGTCACCTAGGCTGGAGTGC-3' |
| RBM3_m^3^C R2 | 5'-GTTGCAGTGAGCTGAGATTGT-3' |
| RBM3_non_m^3^C F | 5'-CTGGTCCCCACCACCTCTAG-3' |
| RBM3_non_m^3^C R | 5'-CAGATCCGTGTGGATCATGC-3' |
| RPS14_m^3^C F | 5'-GTTTGTTACATATGTATACATGTGCCAT-3' |
| RPS14_m^3^C R | 5'-CGAGTTAATGGGTGCAGCAC-3' |
| RPS14_non_m^3^C F | 5'-CCAACATAGCAGCATATGGTG-3' |
| RPS14_non_m^3^C R | 5'-CTTTGCATCCTTCAATGACAC-3' |
| RTN4IP1_m^3^C F | 5'-CAGCCTGGGTGACACAGC-3' |
| RTN4IP1_m^3^C R | 5'-CTTTATTGTCTTTTTTGTTTTAGAGACAGGG-3' |
| RTN4IP1_non_m^3^C F | 5'-GGATCTTTCCCGCATCCAC-3' |
| RTN4IP1_non_m^3^C R | 5'-AAGGAGTCCATTATCGCTGG-3' |
| SDHC_m^3^C F | 5'-CCTCTGGAGTAGCTGGGACTACAG-3' |
| SDHC_m^3^C R | 5'-AAAAATTAGCTGGGCGTAGTGGCG-3' |
| SDHC_non_m^3^C F | 5'-CGGATCCCATTCCAGGTATGATAC-3' |
| SDHC_non_m^3^C R | 5'-TGTTACTCCCTGGGAACTTTGAGTC-3' |
| SENP2_m^3^C F | 5'-CAGCACTTTGGGAGGCCGAG-3' |
| SENP2_m^3^C R | 5'-ACTCCCGACCTCAAGTGATCTG-3' |
| SENP2_non_m^3^C F | 5'-GCAAAGTTTTCTCACAGCAACTGC-3' |
| SENP2_non_m^3^C R | 5'-CACATTTACTCAGCACCAGATGC-3' |
| SOWAHC_m^3^C F1 | 5'-CTGAAAGAGGGTGTGGTGTG-3' |
| SOWAHC_m^3^C R1 | 5'-CCAGACTCAACAAAATCCGATTCAG-3' |
| SOWAHC_m^3^C F2 | 5'-CTGGGTCCCTGAAAGAGGGT-3' |
| SOWAHC_m^3^C R2 | 5'-CGATTCAGAACCCAGATCGTCC-3' |
| SOWAHC_non_m^3^C F | 5'-TTTCCCCGTCACCCTCGTC-3' |
| SOWAHC_non_m^3^C R | 5'-GCCTCCCAGTACCTGAGTCG-3' |
| STX7_m^3^C F | 5'-TTGGGAGGCCAAGGCAGGTA-3' |
| STX7_m^3^C R | 5'-GCTGTTCTCGAACTCCTGACTTC-3' |
| STX7_non_m^3^C F | 5'-CCTTTATAACTTCAGTGGTTCAATC-3' |
| STX7_non_m^3^C R | 5'-CCCTGTGCATCATCATTCTTATC-3' |
| TMX1_m^3^C F | 5'-GACAGGAGTGAAATTTTAAGACCAAAATAAT-3' |
| TMX1_m^3^C R | 5'-TTACAGGCATGTGCCACCATG-3' |
| TMX1_non_m^3^C F | 5'-GCGTTGTCTTATGGCATTCTG-3' |
| TMX1_non_m^3^C R | 5'-GATGTTTCAGAAGAAGAAGCTG-3' |
| TRIM56_m^3^C F | 5'-CTGGAGTGCAGTGGCACAA-3' |
| TRIM56_m^3^C R | 5'-CCAGGAGTTCAAGGCTGCAG-3' |
| TRIM56_non_m^3^C F | 5'-TTGTACTCCCCAACCACCTGG-3' |
| TRIM56_non_m^3^C R | 5'-CATTTCGTGGGGTCGGACTG-3' |
| TRIP11_m^3^C F | 5'-TTAGTAGAGATGGGGTTTCACCA-3' |
| TRIP11_m^3^C R | 5'-GAGTTCAAGACCAGCCTGGC-3' |
| TRIP11_non_m^3^C F | 5'-AACCCCAGCACTGTTGTCAG-3' |
| TRIP11_non_m^3^C R | 5'-GCCCGGGCATCTTCTTCTGAAAC-3' |
| TXNL1_m^3^C F | 5'-CAAACTCCTGACCTCAAGTGATCCA-3' |
| TXNL1_m^3^C R | 5'-CATTTTGGGAGGCCGAGGC-3' |
| TXNL1_non_m^3^C F | 5'-TTTTGCCAACTACTCGTTTGAAG-3' |
| TXNL1_non_m^3^C R | 5'-AGTCGAATCAAGGTGAAGAGG-3' |
| TYRO3_m^3^C F | 5'-AATCAGGAGTTCGAGACCAGC-3' |
| TYRO3_m^3^C R | 5'-CAGGGTTTCGCCATGTTGG-3' |
| TYRO3_non_m^3^C F | 5'-GCAGAGCACCAGCCAGAGAG-3' |
| TYRO3_non_m^3^C R | 5'-CCCTTGCTGCAGCAGCAAAA-3' |
| VPS53_m^3^C F | 5'-TGATCTCGGCTCACTGCAGC-3' |
| VPS53_m^3^C R | 5'-GGAGGATCGCTTGAACCCAG-3' |
| VPS53_non_m^3^C F | 5'-GACAGTGAGCCGGAGCTTTC-3' |
| VPS53_non_m^3^C R | 5'-ACAGAAACCTTTCAGAAGATACTGGACATG-3' |
| WDR12_m^3^C F | 5'-CCAGGATGGAGTGCAATGGC-3' |
| WDR12_m^3^C R | 5'-TGGAGGTTGTGGTGAGCCAA-3' |
| WDR12_non_m^3^C F | 5'-AGATCATAGAGAGGAGCCTTAC-3' |
| WDR12_non_m^3^C R | 5'-GACATCAGTAAAATGGTCTCCTAC-3' |
| ZKSCAN8_m^3^C F | 5'-TCCTTGCCTTCCACCATGATTG-3' |
| ZKSCAN8_m^3^C R | 5'-CTCAGTTCCACATGGCTGGG-3' |
| ZKSCAN8_non_m^3^C F | 5'-GACTTGACCTCTGAATAAAGGC-3' |
| ZKSCAN8_non_m^3^C R | 5'-ATGGGAACACTGGTCTCATTC-3' |
| ZNF100_m^3^C F | 5'-TGCCTGTAATCCCAGAACTTTG-3' |
| ZNF100_m^3^C R | 5'-CCTTAAATGATTTGCCTGCCTC-3' |
| ZNF100_non_m^3^C F | 5'-CTTTTCCTTGCTCCAGACAG-3' |
| ZNF100_non_m^3^C R | 5'-TGTTAGAGAACTACAGAAACCTGG-3' |
| ZNF440_m^3^C F | 5'-ACTAAAAAATACAAAAAATTAGCCTGGCAT-3' |
| ZNF440_m^3^C R | 5'-GTAGCTGGGACTACAGGCAT-3' |
| ZNF440_non_m^3^C F | 5'-ATTCAAAGGTGTGGGGCAGATC-3' |
| ZNF440_non_m^3^C R | 5'-TAAATGCGAGCAATGTGGGAAAGC-3' |
| FAM111A-DT_m^3^C F | 5'-GAAGGGGGAGTGGGCTGTAC-3' |
| FAM111A-DT_m^3^C R | 5'-CATGGTCCTGGGCAGCTCTG-3' |
| FAM111A-DT_non_m^3^C F | 5'-CAGGACCATGGGAACCCAC-3' |
| FAM111A-DT_non_m^3^C R | 5'-ATATGCCTCACATCCAGGTCAC-3' |
| LINC00999_m^3^C F | 5'-TTACTTGCTGGGAGGCAGGG-3' |
| LINC00999_m^3^C R | 5'-AAGTTGTCCTGAAGTCGGCC-3' |
| LINC00999_non_m^3^C F | 5'-GAGAGGCAGGGGCTCATGTC-3' |
| LINC00999_non_m^3^C R | 5'-ACAGTGGCCTGTTGATGCCC-3' |
| lnc-FNBP1L-2_m^3^C F | 5'-TTACGACTCCGCCGGGACTC-3' |
| lnc-FNBP1L-2_m^3^C R | 5'-CTCCGTGGCGCAATGGATAG-3' |
| lnc-FNBP1L-2_non_m^3^C F | 5'-TTACGACTCCGCCGGGACTC-3' |
| lnc-FNBP1L-2_non_m^3^C R | 5'-CGCATTGGACTTCTAGAGGCTG-3' |
| lnc-LINC02203-7_m^3^C R | 5'-ATCAGCTCTGCTCTCTGTGTG-3' |
| lnc-LINC02203-7_non_m^3^C F | 5'-GGCTGAGCCAGGGCTGAGTC-3' |
| lnc-LINC02203-7_non_m^3^C R | 5'-TGGCCATACCATGCCCTGCC-3' |
| lnc-MMD-4_m^3^C F | 5'-CCTCCCAAAGTGCTGGGATTACA-3' |
| lnc-MMD-4_m^3^C R | 5'-CCAGGTTGTGGTGGCTCAC-3' |
| lnc-MMD-4_non_m^3^C F | 5'-ACGGGGTTTCACCATGTTGG-3' |
| lnc-MMD-4_non_m^3^C R | 5'-CGGATCACTTGAGGTCAAGAGTTTG-3' |
| lnc-MPV17L-1_m^3^C F | 5'-AGGCAGATCACGAGGTCAGG-3' |
| lnc-MPV17L-1_m^3^C R | 5'-GTGTTAGCCAGGATGGTCTCA-3' |
| lnc-MPV17L-1_non_m^3^C F | 5'-CGGTGGCTCACACCTGTAATC-3' |
| lnc-MPV17L-1_non_m^3^C R | 5'-CCTCGTGATCTGCCTGCCTC-3' |
| lnc-PRMT9-1_m^3^C F | 5'-ATCACTTGAGGTCAAGAGTTCA-3' |
| lnc-PRMT9-1_m^3^C R | 5'-GTTTCACCATGTTGACCAGG-3' |
| lnc-PRMT9-1_non_m^3^C F | 5'-TTCGGGAGGCCGAAGTGGGT-3' |
| lnc-PRMT9-1_non_m^3^C R | 5'-GGCTGGCCTTGAACTCTTGACCTCAA-3' |
| lnc-TTC5-2_m^3^C F | 5'-ACCAAGCCCGGTTAATTTTTTTTTTGTATTTTAGTA-3' |
| lnc-TTC5-2_m^3^C R | 5'-CCATCCTGGCTAACACGGTG-3' |
| lnc-TTC5-2_non_m^3^C F | 5'-ATGGAGTCTCGTTCTGCCAC-3' |
| lnc-TTC5-2_non_m^3^C R | 5'-TACTGGGGAGGCTGAGGCAG-3' |
| lnc-ZNF280D-3_m^3^C F | 5'-ATGGTGGCACGCACCTGTAA-3' |
| lnc-ZNF280D-3_m^3^C R | 5'-GCCTCAGCCTTCTGAGTAGC-3' |
| lnc-ZNF280D-3_non_m^3^C F | 5'-AACCCAGGAGGCAGAGGTTG-3' |
| lnc-ZNF280D-3_non_m^3^C R | 5'-GTCTGGCTTTGTTGCTCAGG-3' |
| Synthetic RNA sequence used in the *in vitro* methylation assay | |
| Motif sequence | 5'-CUAAUACACCAGUCUUGUAAACCGGAGAUG-3' |

**Supplementary Table 2**

Information on antibodies used in this study.

| Antibody | Source | Catalog Number |
| --- | --- | --- |
| anti-METTL8 | Abcam | ab122273 |
| anti-DDDDK tag | Abcam | ab236777 |
| anti-β-Tubulin | ABclonal | A7196 |
| anti-GAPDH | ABclonal | AC002 |
| anti-m^3^C | Active Motif | 61179 |

**Supplementary Table 3**

Comparison of tRNA m^3^C sites detected by m^3^C-IP-Seq and other methods.

|  |  | **Methods** | | | |  |
| --- | --- | --- | --- | --- | --- | --- |
| **Type** | **Site** | **m^3^C-IP-Seq** | **HAC-seq** | **DM-tRNA-seq** | **HAMR** | **Modomics data** |
| ArgCCU | C32 | √ | √ | √ |  | √ |
| ArgUCU | C32 | √ | √ | √ | √ | √ |
| LeuCAG | C47d | √ | √ | √ |  | √ |
| MetCAU | C20 | √ | √ | √ | √ | √ |
| SerAGA | C32 | √ | √ | √ |  | √ |
| SerAGA | C47d |  | √ | √ |  | √ |
| SerCGA | C32 | √ | √ | √ | √ | √ |
| SerCGA | C47d |  | √ | √ |  | √ |
| SerGCU | C32 | √ | √ | √ |  |  |
| SerGCU | C47d | √ | √ | √ |  | √ |
| SerUGA | C32 | √ | √ | √ | √ | √ |
| SerUGA | C47d |  | √ | √ |  | √ |
| ThrAGU | C32 | √ | √ | √ | √ | √ |
| ThrCGU | C32 | √ | √ | √ | √ |  |
| ThrUGU | C32 | √ | √ | √ | √ | √ |
| mt-MetCAU | C32 |  |  |  | √ |  |
| mt-Ser2UGA | C32 | √ | √ | √ | √ |  |
| mt-ThrUGU | C32 | √ | √ | √ | √ |  |

**Supplementary Figures**

**
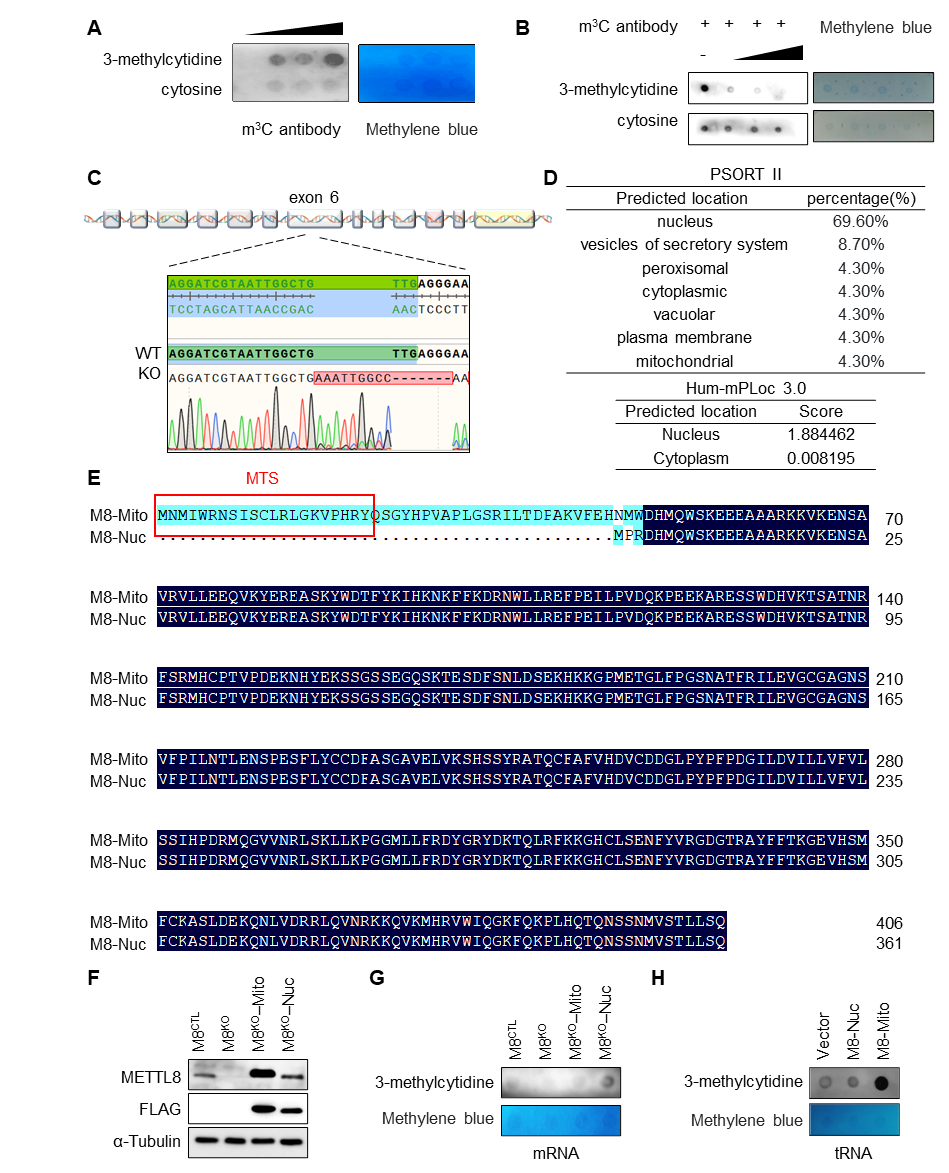
**

**Figure S1. m^3^C modifications present in mRNAs.**

(A) Dot blot using anti-m^3^C antibody against oligonucleotides containing either m^3^C or unmodified cytosine. Loading was visualized by methylene blue staining. (B) A competitive dot blot assay was conducted on membranes spotted with tRNAs. Anti-m^3^C antibody binding to tRNAs was attenuated by preincubation with increasing amounts of 3-methylcytidine (top), but not with cytosine (bottom). Methylene blue staining (right) was included for loading control. (C) Schematic representation of the genomic locus targeted for CRISPR/Cas9-mediated *METTL8* knockout. sgRNA was designed to target Human *METTL8* exon 6 (top). The *METTL8* KO cell was verified to have 2 extra bases resulting in a rearrangement of the amino acid sequence of METTL8. (D) Predicted subcellular localization of METTL8 isoform (NP_001308087.1) using PSORT II and Hum-mPLoc 3.0. (E) Sequence alignment of human M8-Mito (UniProt: B3KW44) and M8-Nuc (UniProt: B4DLT0). The predicted mitochondrial targeting signal (MTS) sequence was highlighted in the red box. (F) Immunoblot analysis of M8^CTL^ and M8^KO^ HEK293T cells with or without expressing FLAG-tagged M8-Mito/M8-Nuc. α-Tubulin was included for loading control. (G) Dot blot using anti-m^3^C antibody against mRNAs from M8^CTL^ and M8^KO^ HEK293T cells with or without expressing FLAG-tagged M8-Mito/M8-Nuc. Loading was visualized by methylene blue staining. (H) Dot blot using anti-m^3^C antibody against tRNAs from HEK293T cells expressing FLAG-tagged M8-Mito/M8-Nuc. Loading was visualized by methylene blue staining.


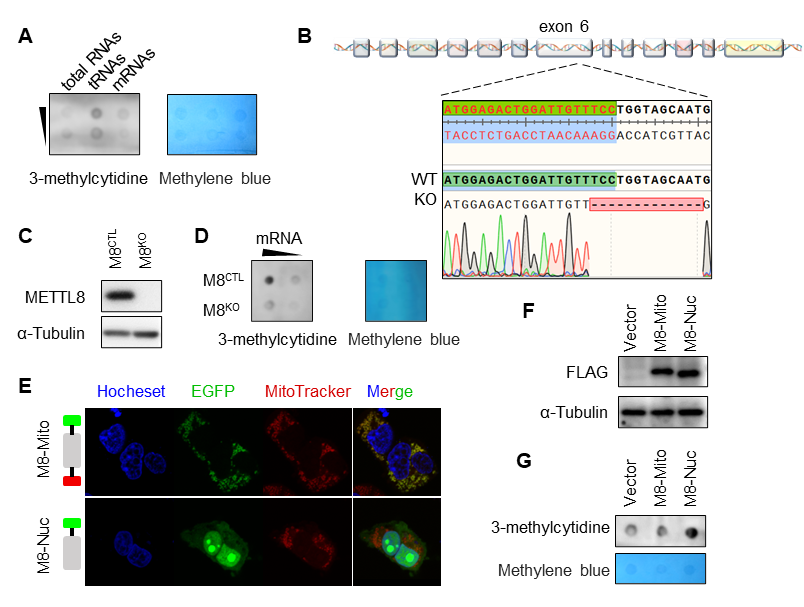


**Figure S2. METTL8-Nuc mediates m^3^C methylation formation in mRNAs in HCT116 cells.**

(A) Dot blot using anti-3-methylcytidine (m^3^C) antibody (left) against total RNAs, tRNAs, and mRNAs from wild-type HCT116 cells. Methylene blue staining (right) was included for loading control. (B) Schematic representation of the genomic locus targeted for CRISPR/Cas9-mediated *METTL8* knockout. sgRNA was designed to target human *METTL8* exon 6. The *METTL8* KO cell was verified to have a 13-base deletion, resulting in a rearrangement of the amino acid sequence of METTL8. (C) Immunoblot analysis of wild-type HCT116 cells (M8^CTL^) or *METTL8* knockout cells (M8^KO^). α-Tubulin was included as a loading control. (D) Dot blot using anti-m^3^C antibody against mRNAs from M8^CTL^ and M8^KO^ HCT116 cells. Loading was visualized by methylene blue staining. (E) Confocal microscopy images of HCT116 cells expressing EGFP-tagged M8-Mito or M8-Nuc. The nuclei and mitochondria were stained with Hoechst and MitoTracker, respectively. Construct schematics were on the left. (F) Immunoblot analysis of HCT116 cells expressing FLAG-tagged M8-Mito/M8-Nuc. α-Tubulin was included for loading control. (G) Dot blot using anti-m^3^C antibody against mRNAs from HCT116 cells expressing FLAG-tagged M8-Mito/M8-Nuc. Loading was visualized by methylene blue staining.


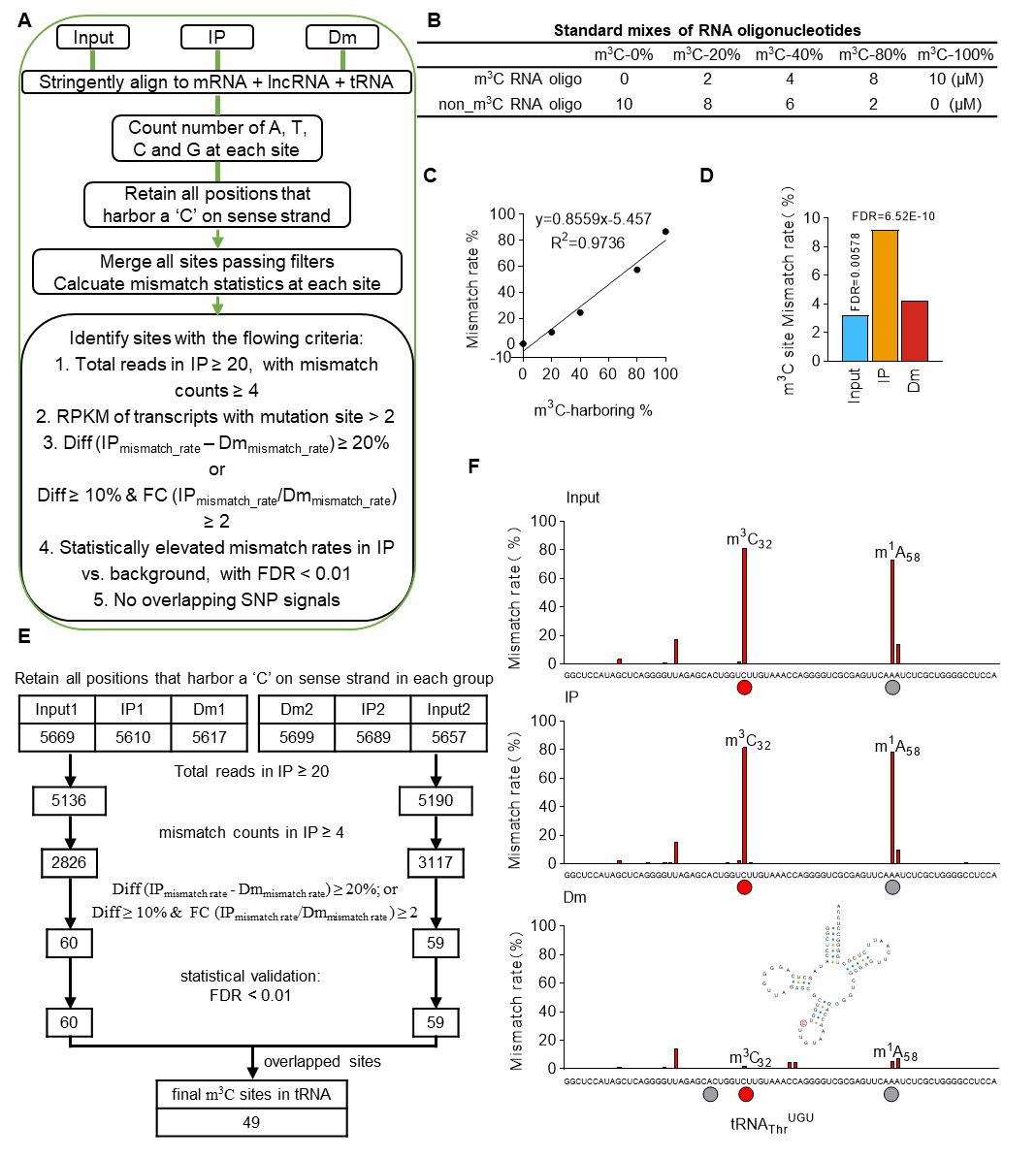


**Figure S3. Analytical pipeline and performance of m^3^C-IP-Seq for identification of m^3^C sites.**

(A) Overview of the analytical pipeline applied to the three data sets. (B) Mixtures of m^3^C RNA oligo (in micromoles) and non_m^3^C RNA oligo (in micromoles) with different concentrations. (C) A linear relationship between the theoretical proportion of m^3^C and the mismatch rate detected by m^3^C-IP-seq. (D) The changes in misincorporation rates detected by m^3^C-IP-seq after the addition of a 10% m^3^C-harboring spike-in to the Input group were analyzed by using statistical testing to identify m^3^C sites with significantly elevated mismatch rates in the IP group compared to the background mutation rate derived from the Dm group. (E) Flowchart detailing the analytic workflow for identifying m^3^C-containing sites in tRNAs. The number of sites passing each filtering criterion is annotated in the figure. (F) Identification of an m^3^C site at position 32 in the cytosolic human tRNA_Thr_^UGU^ by m^3^C-IP-seq. Two other modifications (at positions 26 and 58) were not sensitive to antibody enrichment or demethylation.


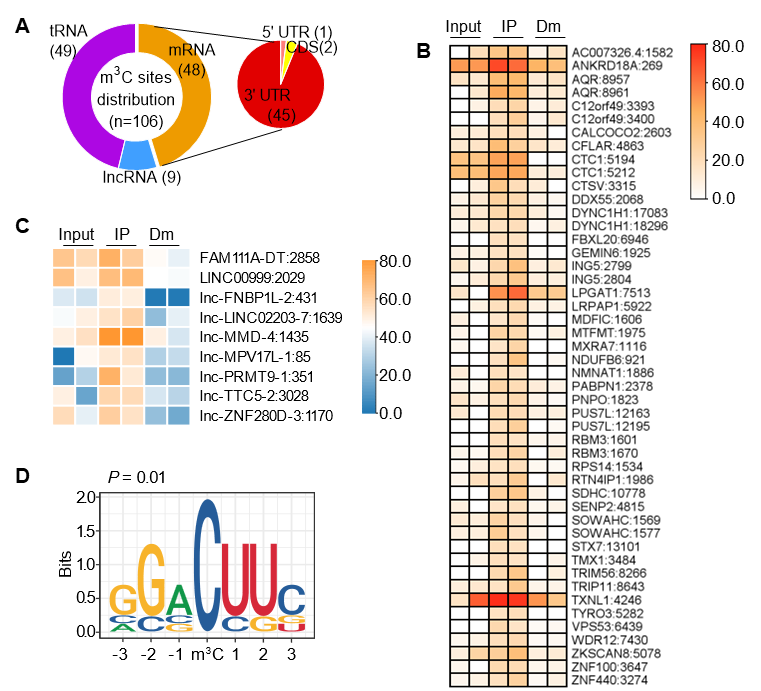


**Figure S4. Single-nucleotide resolution m^3^C methylome in the human transcriptome.**

(A) Pie chart showing the distribution of m^3^C sites identified in different RNA species within the human transcriptome. (B, C) Heat maps showing the mismatch rates for all m^3^C-modified mRNA sites (B), and lncRNA sites (C) identified from m^3^C-IP-seq datasets. Mismatch rates were shown for Input, IP, or Dm samples of two replicates. (D) Consensus motif of m^3^C sites in lncRNAs.


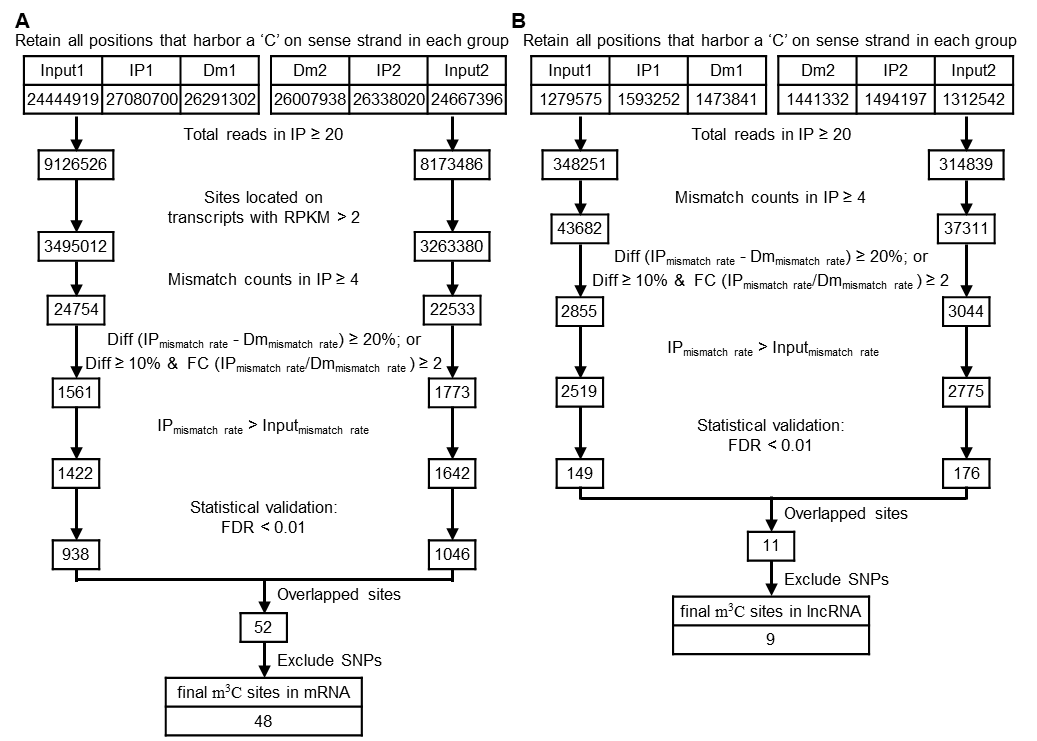


**Figure S5. Detailed information on the number of sites passing each filtering criterion for mRNAs and lncRNAs**

(A, B) Flowcharts detailing the analytic workflow for identifying m^3^C-containing sites in mRNAs (A) and lncRNAs (B). The number of sites passing each filtering criterion is annotated in the figures.


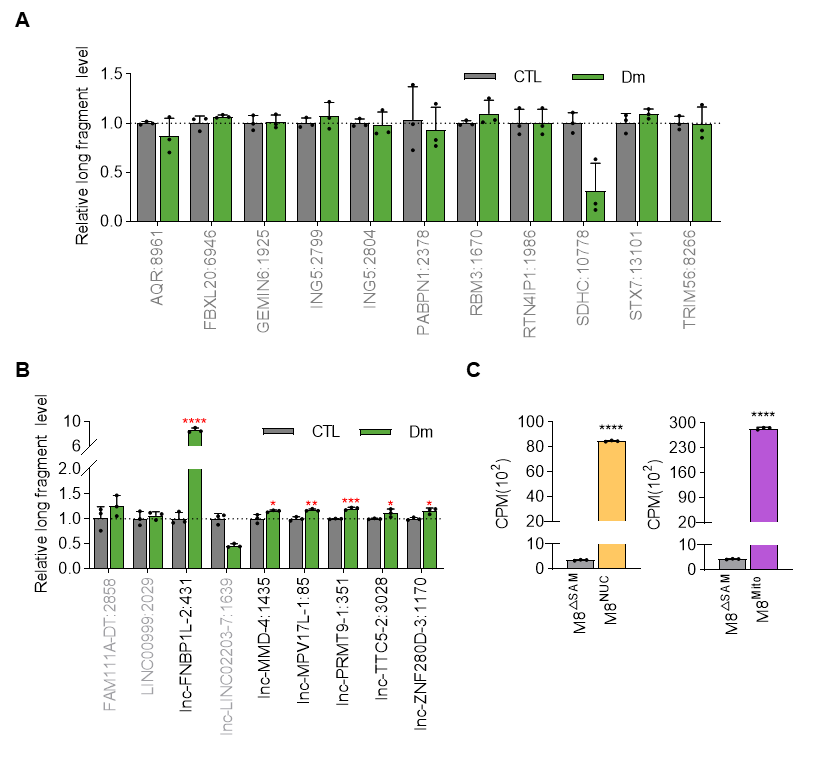


**Figure S6. Validation of m^3^C in mRNAs and lncRNAs.**

(A, B) Relative long fragment expressions of mRNA (A) and lncRNA (B) m^3^C sites with or without demethylation detected by m^3^C-truncated-qPCR. Data were normalized to the value of M8^CTL^. Values represent mean ± SEM (**P*<0.05, ***P*<0.01, ****P*<0.001, *****P*<0.0001; 3 independent experiments, *t*-test, two-tailed). Successfully validated sites were shown in black, while unsuccessfully validated sites were shown in gray. (C) The identified m^3^C motif was used for *in vitro* methylation assays with purified METTL8 proteins and [^3^H]-SAM. Tritium incorporation into RNA was measured by scintillation counting. The bar plot shows mean counts per minute (CPM) from n = 3 independent experiments ± SEM (*****P* < 0.0001; *t*-test, two-tailed).
